# The genome of the yellow potato cyst nematode, *Globodera rostochiensis*, reveals insights into the basis of parasitism and virulence

**DOI:** 10.1186/s13059-016-0985-1

**Published:** 2016-06-10

**Authors:** Sebastian Eves-van den Akker, Dominik R. Laetsch, Peter Thorpe, Catherine J. Lilley, Etienne G. J. Danchin, Martine Da Rocha, Corinne Rancurel, Nancy E. Holroyd, James A. Cotton, Amir Szitenberg, Eric Grenier, Josselin Montarry, Benjamin Mimee, Marc-Olivier Duceppe, Ian Boyes, Jessica M. C. Marvin, Laura M. Jones, Hazijah B. Yusup, Joël Lafond-Lapalme, Magali Esquibet, Michael Sabeh, Michael Rott, Hein Overmars, Anna Finkers-Tomczak, Geert Smant, Georgios Koutsovoulos, Vivian Blok, Sophie Mantelin, Peter J. A. Cock, Wendy Phillips, Bernard Henrissat, Peter E. Urwin, Mark Blaxter, John T. Jones

**Affiliations:** Division of Plant Sciences, College of Life Sciences, University of Dundee, Dundee, DD1 5EH UK; Institute of Evolutionary Biology, University of Edinburgh, Edinburgh, EH9 3FL UK; Cell and Molecular Sciences Group, Dundee Effector Consortium, James Hutton Institute, Dundee, DD2 5DA UK; Centre for Plant Sciences, University of Leeds, Leeds, LS2 9JT UK; INRA, University Nice Sophia Antipolis, CNRS, UMR 1355-7254 Institut Sophia Agrobiotech, 06900 Sophia Antipolis, France; Wellcome Trust Sanger Institute, Wellcome Trust Genome Campus, Cambridge, CB10 1SA UK; School of Biological, Biomedical and Environmental Sciences, University of Hull, Hull, HU6 7RX UK; INRA, UMR1349 IGEPP (Institute for Genetics, Environment and Plant Protection), 35653 Le Rheu, France; Agriculture and Agri-food Canada, Horticulture Research and Development Centre, 430 Bboul. Gouin, St-Jean-sur-Richelieu, Quebec, J3B 3E6 Canada; Sidney Laboratory, Canadian Food Inspection Agency (CFIA), 8801 East Saanich Rd, Sidney, BC V8L 1H3 Canada; Laboratory of Nematology, Department of Plant Sciences, Wageningen University, Droevendaalsesteeg 1, 6708 PB Wageningen, The Netherlands; Information and Computational Sciences Group, James Hutton Institute, Dundee, UK; USDA-ARS Horticultural Crops Research Laboratory, Corvallis, OR USA; CNRS UMR 7257, INRA, USC 1408, Aix-Marseille University, AFMB, 13288 Marseille, France; Department of Biological Sciences, King Abdulaziz University, Jeddah, Saudi Arabia; School of Biology, University of St Andrews, North Haugh, St Andrews KY16 9TZ UK

**Keywords:** Plant-parasitic nematode, Genome sequence, Virulence, Effectors, Horizontal gene transfer

## Abstract

**Background:**

The yellow potato cyst nematode, *Globodera rostochiensis*, is a devastating plant pathogen of global economic importance. This biotrophic parasite secretes effectors from pharyngeal glands, some of which were acquired by horizontal gene transfer, to manipulate host processes and promote parasitism. *G. rostochiensis* is classified into pathotypes with different plant resistance-breaking phenotypes.

**Results:**

We generate a high quality genome assembly for *G. rostochiensis* pathotype Ro1, identify putative effectors and horizontal gene transfer events, map gene expression through the life cycle focusing on key parasitic transitions and sequence the genomes of eight populations including four additional pathotypes to identify variation. Horizontal gene transfer contributes 3.5 % of the predicted genes, of which approximately 8.5 % are deployed as effectors. Over one-third of all effector genes are clustered in 21 putative ‘effector islands’ in the genome. We identify a dorsal gland promoter element motif (termed DOG Box) present upstream in representatives from 26 out of 28 dorsal gland effector families, and predict a putative effector superset associated with this motif. We validate gland cell expression in two novel genes by in situ hybridisation and catalogue dorsal gland promoter element-containing effectors from available cyst nematode genomes. Comparison of effector diversity between pathotypes highlights correlation with plant resistance-breaking.

**Conclusions:**

These *G. rostochiensis* genome resources will facilitate major advances in understanding nematode plant-parasitism. Dorsal gland promoter element-containing effectors are at the front line of the evolutionary arms race between plant and parasite and the ability to predict gland cell expression a priori promises rapid advances in understanding their roles and mechanisms of action.

**Electronic supplementary material:**

The online version of this article (doi:10.1186/s13059-016-0985-1) contains supplementary material, which is available to authorized users.

## Background

All major crops are thought to be infected by at least one species of plant-parasitic nematode, which causes damage valued at over $80 billion each year [1]. The majority of these economic losses are attributable to the sedentary endoparasitic nematodes of the genus *Meloidogyne* (root-knot nematodes) and the genera *Heterodera* and *Globodera* (cyst nematodes). These sedentary endoparasites have complex biotrophic interactions with their hosts that include induction of specific feeding sites and long residence times within or on their host(s).

Potato cyst nematodes (PCN) are economically important pathogens of potato, with two major species: the white PCN *Globodera pallida* and the yellow PCN *G. rostochiensis*. These nematodes originate in South America [2, 3] and have subsequently been introduced into all major potato-growing regions of the world. Europe has acted as a secondary distribution hub for PCN; worldwide populations outside South America reflect subsequent introductions from Europe [4, 5]. Once established in a field, PCN are effectively impossible to eradicate in the short term and because they persist as long-lived cysts in soils, growing potatoes may not be economically viable for up to two decades. As a result, the US Department of Agriculture (USDA) has classified the yellow PCN as potentially more dangerous than any insect or disease affecting the potato industry (Aphis USDA 12/09/2015). Substantial effort is thus invested into keeping land free of PCN; both species are present on USDA and European Plant Protection Organisation quarantine organism lists.

PCN have been classified to pathotype based on their relative virulence on host plants harbouring different resistance loci. Most of the *G. rostochiensis* in UK potato-growing regions is of pathotype Ro1 and can be controlled by a single major resistance locus (H1). UK *G. rostochiensis* populations have therefore been suggested to originate from a genetically restricted introduction into Europe [6, 7]. Other pre-existing *G. rostochiensis* pathotypes (Ro 2, 3 and 5, but not 4) are able to overcome H1 resistance [8] and these pathotypes may be selected in response to widespread deployment of H1 plants. The corresponding nematode avirulence gene(s) has not been identified. Understanding the bases of virulence and resistance is of critical importance for agriculture.

*G. rostochiensis* has a complex life cycle that includes a highly resistant survival stage. Cysts, formed from the body wall of the adult female, encase hundreds of eggs that can lie dormant in the soil for over 20 years. Second stage juveniles (J2) within the eggs hatch in response to root diffusates from suitable host plants growing nearby. The J2 nematodes locate the root and migrate destructively through root tissues until they reach the inner cortex layers. Here the nematodes probe the cells, until a cell that does not respond adversely is detected [9]. This initial syncytial cell is transformed into a large, multinucleate syncytium in response to proteins, peptides and hormones secreted by the nematode. Cell wall openings are formed between the initial syncytial cell and its neighbours, followed by fusion of the protoplasts. Syncytial cells become highly metabolically active and have enriched cytoplasm, enlarged nuclei and a greatly reduced central vacuole. Additional layers of cells are subsequently incorporated into the syncytium, which may eventually be composed of up to 300 cells [9]. A prolonged biotrophic interaction is then maintained for a period of several weeks, while the nematode intermittently withdraws host cytoplasm to derive all food required for development to the adult stage. Each nematode can only induce a single feeding site that must therefore be maintained and protected from host defences.

The complex interactions of PCN with their hosts, like those of other plant parasites and pathogens, are mediated by effectors: secreted proteins that manipulate the host to the benefit of the pathogen. Most PCN effectors are produced in two sets of gland cells, dorsal and subventral [10], although some apoplastic effectors can be produced in the gland cells surrounding the main anterior sensory organs, the amphids [11]. Effectors play important roles in all aspects of the parasite-host interaction: invasion and migration [12], suppression of host defences [13] and induction of the feeding site [14, 15]. The effector repertoire of plant-parasitic nematodes, including PCN, has been augmented by multiple Horizontal Gene Transfer (HGT) events, primarily of bacterial origin [16]. HGT events are suspected to have played an important role in the emergence of plant parasitism in nematodes, enabling degradation of the plant cell wall, nutrient processing and manipulation of plant defences [17]. Due to their importance in the life cycle of plant-parasitic nematodes, a great deal of effort has been put into various approaches for effector identification, including genomic and transcriptomic analyses [10], transcriptomic analyses of purified gland cells [18] and proteomic analyses [19]. For some effectors, the likely biological functions, including host proteins targeted, have been identified [14, 20, 21].

Here, we report a high quality draft genome of a Ro1 isolate of *G. rostochiensis*, in combination with replicated transcriptome data from four key life stages, and genome sequence from eight populations across four pathotypes. We conducted whole genome comparisons between *G. rostochiensis* and related species [22–25] to explore the genomic and transcriptomic bases of pathogenicity. We discovered an unusually high frequency of well-supported non-canonical splice sites in *G. rostochiensis*, and found that this phenomenon was also present in related parasitic nematode species. Using an HGT analysis pipeline, we identified hundreds of genes in the *G. rostochiensis* genome that may have been acquired by gene transfers from non-metazoan origin, some of which likely play important roles in plant parasitism. We identified effectors in *G. rostochiensis* and found that they frequently grouped together into ‘effector islands’. To explore the genetic bases of virulence, we compared genetic variation in effectors and other genes between pathotypes and found that effectors, in general, contained more non-synonymous mutations. Using the identified *G. rostochiensis* effectors as a training set, we identified a putative ‘DOrsal Gland promoter element’, or DOG box, which was also associated with effectors in related species. We were able to use the DOG box to predict novel effectors, confirmed by in situ hybridisation, in *G. rostochiensis*, and to identify all putative DOG effectors from available cyst nematode genomes.

## Results and discussion

### The genome sequence of *Globodera rostochiensis* Ro1

The genome of the potato cyst nematode, *G. rostochiensis*, pathotype Ro1 from the James Hutton Institute collection, was sequenced to 435.6-fold coverage and assembled into a high quality draft assembly (nGr.v1.0) of 95.9 Mb (Table 1), consistent with experimental estimates of *Globodera* genome size [26]. The assembly shows a smaller size and total gene number, yet higher completeness than the *G. pallida* genome [22] (Table 1). Further, the low level of duplication of core, conserved genes (Table 1), and indeed of all genes (Additional file 1: Figure S1), suggest that the *G. rostochiensis* genome assembly is a more accurate representation of a *Globodera* genome, probably reflective of the low genetic variation present in the UK *G. rostochiensis* used for sequencing [11].

**Table 1:** Genome statistics

	*G. pallida*	*G. rostochiensis*
Assembly version	nGp.v1.0	nGr.v1.0
Assembly size (Mb)	124.6	95.9
Scaffolds (n)	6873	4377
Scaffold N50 (bp)	121,687	88,495
Longest scaffold (bp)	600,076	688,384
Contig N50 (bp)	11,611	11,371
Longest contig (bp)	93,564	111,501
Span of N’s in assembly (bp)	21,024,229	4,445,051
GC (%)	36.7	38.1
CEGMA (Complete/Partial %)	74.19/80.65	93.55/95.56
Average CEG gene number (Complete/Partial)	1.23/1.29	1.15/1.24
Gene density (per Mb)	132.2	149.9
Genes (n)	16,466	14,378
Proteins (n)	16,417	14,309
Proteins w/Start and Stop codon (n)	14,580 (88.81 %)	13,083 (91.43 %)
Non-canonical splice sites (%)	3.56 % (n = 4059)	3.46 % (n = 3835)
PfamA domains (cutoff 1e-5) (n)	8853	8397
Best BLAST hit to nematode proteins (1e-10) (n)	8886	8603

### Collaborative manual gene refinement reveals a uniquely high frequency of non-canonical splice sites in *Globodera*

**Fig. 1:**
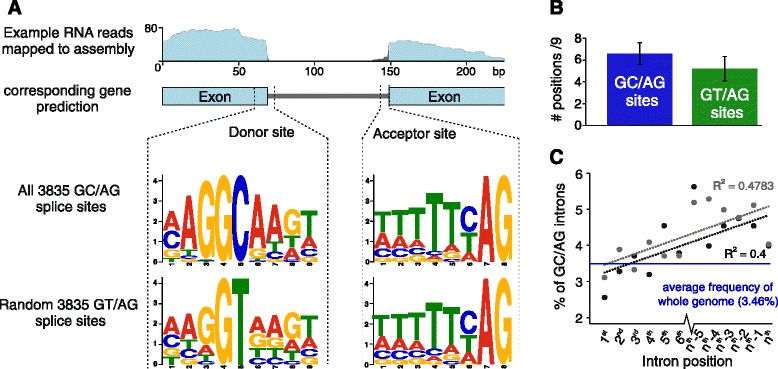
Non-canonical splicing in Globodera. **a** Correction of many exon-intron boundaries to be consistent with RNA-seq mapping required the use of a non-canonical 5′ donor site. Comparison of the consensus sequence for both canonical (GT/AG) and non-canonical (GC/AG) splice sites reveals similar local base composition, with the exception of the GT or GC itself. **b** The 5′ donor sites of both GC/AG and GT/AG introns conform to the consensus CAGG[T|C]AAGT. **c** GC/AG introns are less common at the beginning of gene models in both *G. rostochiensis* (*black*) and *G. pallida* (*grey*)

To produce a high quality set of gene predictions, an initial phase of automated annotation was followed by manual refinement of approximately one-eighth of all gene models in the collaborative genome annotation editor WebApollo (Additional file 2: Supplementary information file 1). During the manual annotation phase, we noted that correction of many exon-intron boundaries to be consistent with mapped RNA-sequencing (RNA-seq) data (Fig. 1a) was only possible using non-canonical 5′ donor splice sites (GC rather than GT). The frequency of GC-bearing introns in the manual annotation set was two orders of magnitude higher than in the initial automated predictions. However, genome-wide re-prediction, using manually curated genes as a training set and allowing for the prediction of non-canonical GC/AG introns, increased the frequency of GC/AG introns to that of the manually annotated set (Additional file 3: Table S1) and markedly improved upon automated predictions (see Additional file 2: Supplementary information file 1).

The frequency of GC/AG introns in *G. rostochiensis* was 3.46 %, the highest reported for any nematode. In addition to the GT or GC dinucleotide, 5′ donor sites are characterised by a nine-base consensus sequence, CAGG[T|C]AAGT (where the initial CAG is in the preceding exon [27]). Although variations in the 5′ donor site sequence were found, *G. rostochiensis* GC/AG introns conformed equally well, if not better, to this consensus as did GT/AG introns (Fig. 1a and b). We derived a revised 5′ donor consensus for the predicted introns for both GC and GT 5′ sites and found both intron classes to use AAGG[T|C]AAGT (where the first AAG is in the preceding exon). We identified a similarly high frequency of GC/AG introns in *G. pallida* (3.53 %), and *Rotylenchulus reniformis* (2.36 %) (PRJNA214681, Showmaker et al., unpublished), a sedentary endoparasite of multiple crop plants that is in a sister group to *Globodera* in the Tylenchoidea (Additional file 4: Figure S2). While GC/AG introns were apparently absent from the *Meloidogyne* species gene predictions, we suspect this may be due to restrictive settings during their annotation, as they are present in most species (Additional file 4: Figure S2). The elevated proportion of non-canonical GC/AG introns appear to be restricted to the Heteroderidae.

In species pairs with a low GC/AG intron frequency, such as *Caenorhabditis elegans* and the closely related *C. briggsae*, there is no obvious conservation of non-canonical splice site usage in their orthologous genes [28]. However, for genes in *G. rostochiensis* with at least one GC/AG intron, ~72 % of the corresponding one-to-one orthologues in *G. pallida* also contained at least one GC/AG intron (n = 2148), compared to an average of 10.8 % for identically sized subsets of non-GC/AG intron containing *G. rostochiensis* genes (1000 iterations, stdev = 0.8 %). Within those genes, orthologous introns also tended to have conserved non-canonical splice sites. For 30 % of the *G. rostochiensis* GC/AG introns in one-to-one orthologues, the corresponding *G. pallida* intron also used GC/AG. GC/AG introns had a biased distribution within genes in both species, tending to be less common in introns in the 5′ portion of genes compared to introns in the 3′ portion (Fig. 1c).

### Life stage specific transcriptome

**Fig. 2:**
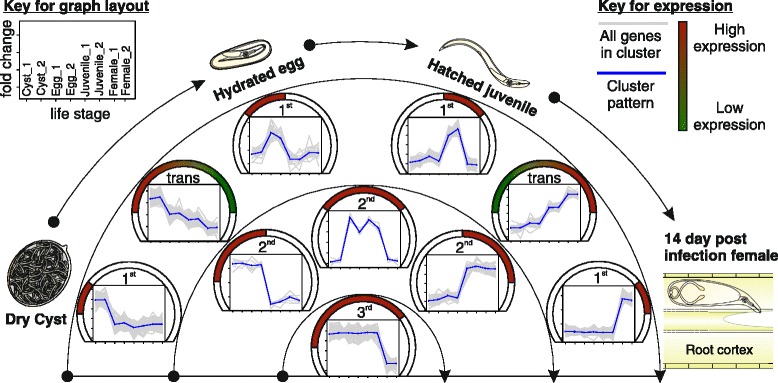
Example of differential gene expression clusters in the context of nematode biology. The transcriptome of *G. rostochiensis* was sequenced in duplicate for four key stages across the life cycle: dormant cysts; hydrated eggs; hatched infective juveniles (J2); and feeding 14 days post infection (dpi) females. A subset of the 6720 genes differentially expressed (FDR <0.001, min fold 4) are grouped into expression clusters which describe the genes specifically upregulated at various life stages. Clusters which uniquely describe each life stage (1st order), describe two life stages (2nd order) or describe three of the four life stages (3rd order) are identified. Further, some expression clusters show a stepwise increase (or decrease) in expression as the nematode transitions through its life cycle (trans). For all expression clusters, mean centred log fold-change of expression is plotted for each of two biological replicates for each life stage in the following order: Cyst, egg, J2, 14 dpi female. All genes in each cluster are drawn with *grey bars*, the average of which is shown in *blue*

From the *G. pallida* genome project [22], it was clear that the key parasitic transitions to be captured in terms of all cyst nematode gene expression, and in particular for effectors, is from outside the plant (J2) compared to inside the plant (sedentary females). We used nematodes at 14 days post infection (dpi) as this provides an ideal intermediate for the sedentary stages: variation in gene expression at 14 dpi accounts for most of the variation in gene expression at 7 dpi (84 %), and at 21 dpi (60 %, Additional file 5: Figure S3). *G. rostochiensis* pathotype Ro1 gene expression was therefore analysed at four key stages across the life cycle: dormant cysts; hydrated eggs; hatched infective J2; and feeding parasitic females. Using a false discovery rate (FDR) of <0.001 and a minimum fold-change of 4, 6720 genes (47 %) were found to be differentially expressed. Differentially expressed genes were grouped into expression clusters; those that uniquely describe each life stage, two life stages or three life stages were identified (Fig. 2; expanded in Additional file 6: Figure S4; Additional file 7: File S1 contains the data matrix of normalised expression values). Some expression clusters showed a stepwise increase or decrease in expression corresponding to transitions through the life cycle. As much as 94 % of all differentially expressed genes, and thus ~44 % of all genes, are manually grouped into 25 biologically relevant expression super-clusters (Additional file 6: Figure S4).

**Fig. 3:**
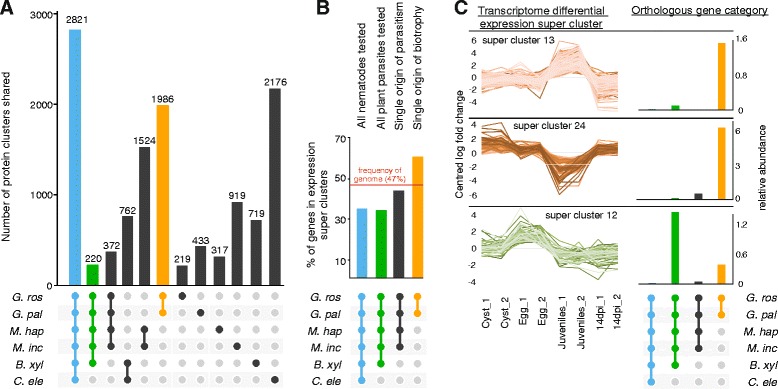
Putative orthologous gene clusters in related nematode species and transcriptomic analysis. **a** The predicted protein sets of five plant parasites, from two independent origins of parasitism, were compared to that of the free living nematode *C. elegans*. The analysis includes two cyst nematodes, *G. rostochiensis* and *G. pallida*, two root knot nematodes, *M. hapla* and *M. incognita*, the pine wilt nematode *B. xylophilus* and the free living nematode *C. elegans*. The *histogram* shows the number of clusters shared uniquely between the species highlighted below. A total of 1986 clusters of genes are present in both cyst nematode species and absent from all other nematodes tested (*orange*). **b** Focusing on four orthologous gene cluster categories (all nematodes, all plant parasites, a single origin of plant parasitism and a single origin of sedentary biotrophy), the percentage of genes present in differential super-clusters suggest that those genes unique to the Globodera are preferentially differentially regulated throughout the adapted life cycle. **c** Analysing the relative abundance of genes from each orthologous gene cluster category in each of the differential expression super-clusters suggests that those genes specifically differentially regulated during the infective juvenile stage are those unique to the Globodera

*G. rostochiensis* predicted proteins were clustered with those from the cyst nematode *G. pallida* [20], the root-knot nematodes *M. hapla* [24] and *M. incognita* [23], the pine wilt nematode *Bursaphelenchus xylophilus* [29] and *C. elegans* (Fig. 3a; for relationships between these species see Additional file 4: Figure S2). Among the 16,821 OrthoMCL clusters, 2821 contained representatives from all nematodes tested, 220 clusters contained only proteins from plant parasites, 372 clusters contained only proteins of *Globodera* spp. and *Meloidogyne* spp. and 1986 clusters were composed solely of proteins from the cyst nematodes *G. rostochiensis* and *G. pallida*.

Focusing on these four categories of orthologous clusters (all nematodes, all plant parasites, *Meloidogyne* plus *Globodera* and *Globodera*) we correlated the orthologue definition and transcriptional clustering data to explore possible functional roles of genes unique to subsets of the taxa analysed. Only 34 % of genes in clusters with members from all five nematodes, or clusters lacking only *C. elegans*, were differentially expressed, compared to 47 % differentially expressed overall (Fig. 3b), congruent with the assumption that these families are likely to include loci with roles in core physiology. Interestingly however, genes specifically upregulated in eggs contain a higher relative abundance of genes in orthologous clusters common to all plant parasites yet absent in *C. elegans*, compared to other orthologous gene categories (Fig. 3c).

Only 43 % of genes in orthologous clusters private to *Meloidogyne* and *Globodera* were differentially expressed. In contrast, of the genes in orthologous clusters only present in the two *Globodera* species, 60 % were differentially regulated, suggesting that these genes play a dynamic role in parasite development. Furthermore, over two-fifths of genes (42 %) that are differentially regulated in the infective juvenile stage of *G. rostochiensis* are those that are unique to the *Globodera*. Expression super-clusters 13 and 24, which describe those genes specifically upregulated or downregulated in the infective juvenile stage, respectively, contain a higher relative abundance of genes in orthologous clusters unique to *Globodera* species compared to other orthologous gene categories (Fig. 3c).

*G. rostochiensis* proteins in clusters private to *Meloidogyne* and *Globodera* were enriched for GO terms associated with gene silencing by miRNA (*p* <0.001, FDR 0.05), including nine proteins with highest similarity to worm-specific argonautes (WAGOs) in *C. elegans*. WAGOs are central to the RNAi pathway, being responsible for binding of small RNAs and mediation interactions with other proteins, and show an exceptional diversity within the phylum Nematoda. It has been suggested that the expansion of WAGOs within Nematoda is associated with extreme functional plasticity [30]. Enrichment of WAGOs in the *Meloidogyne* and *Globodera* lineage, in combination with phylogenetically distinct clades of WAGOs in the Heteroderidae (Additional file 8: Figure S5), may indicate functional diversification following expansion. With the exception of GROS_g08854, all *G. rostochiensis* WAGOs that are differentially regulated are present in differential expression super-clusters 19, 20 and 21. All but one of these differentially expressed WAGOs are in Clades 1/2/4/5 and 10/11. Expression super-clusters 19, 20 and 21 are characterised by significant upregulation at 14 dpi, suggesting a dynamic role for WAGO clade 1/2/4/5 and 10/11 as *G. rostochiensis* transitions through parasitism.

### Genes acquired by horizontal transfer have substantially contributed to the genome of *G. rostochiensis*

Horizontal gene transfer (HGT) events have played an important role in the emergence of plant-parasitism in nematodes [17]. Numerous plant cell wall degrading enzymes, originally acquired from bacteria, are present in a wide range of tylenchomorph plant-parasitic nematode species, while diplogasterid nematodes have acquired functionally analogous genes from fungi [17]. Using a systematic genome-wide approach, putative HGT events were identified based on the ratio of their sequence similarity to metazoan and non-metazoan sequences (Alien Index (AI), (Alienness [31–33])). Proteins with an AI >0 and more than 70 % identity to a non-metazoan sequence were considered putative contaminants (*n* = 18) and not included in these analyses.

We identified 519 *G. rostochiensis* proteins that may have originated through HGT events (AI >0), including all previously published cases of HGT into cyst nematodes present in the predicted proteins (Table 2). Of the 519 genes putatively acquired by HGT, 87 % have some evidence of transcription at the four life stages sampled (cumulative FPKM > 1, *c.f.* 95 % of all proteins), 91 % have at least one intron (*c.f.* 95 % of all proteins) and 92 % are on scaffolds containing other genes not predicted to be acquired by HGT (*c.f.* 95 % of random set (n = 519), 1000 iterations). We found strong support (AI >30) for 91 proteins (Additional file 9: Table S2). In 77 % of these cases (70/91), the most similar sequences identified were of bacterial origin, while in ~11 % (10/91), the most similar sequences were of fungal origin, consistent with previous reports of HGT in plant-parasitic nematodes. The remaining proteins with an AI >30 had closest similarity to proteins from protists (*n* = 7), plant (*n* = 3) and a virus (*n* = 1). No phylogenetically confirmed HGT of protist, plant or virus origin has been identified to date in plant-parasitic nematodes. Given that some of these candidates are among genes with evidence of expression, they deserve further investigation.

**Table 2:** Genes acquired via HGT in other cyst and root-knot nematodes also found in the genome of *G. rostochiensis*

Process	Gene family	Function	Pfam domains	Highest AI	Reference	*G. rostochiensis* genes
Cell wall degradation	GH5_2 Cellulases	Cellulose degradation	PF00150 Cellulase (glycosyl hydrolase family 5)	198.94	[12]	GROS_g01454
						GROS_g04677
						GROS_g05961
						GROS_g05962
						GROS_g07338
						GROS_g07446
						GROS_g07949
						GROS_g10505
						GROS_g11008
						GROS_g11200
						GROS_g11949
	Expansin-like proteins	Softening of non-covalent bonds	PF03330 Rare lipoprotein A (RlpA)-like double-psi beta-barrel	29.93	[90]	GROS_g03476
						GROS_g09961
						GROS_g10585
						GROS_g11726
						GROS_g11727
						GROS_g12817
						GROS_g12966
	GH53 candidate Arabinogalactan endo-1,4-beta-galactosidase	Pectinose/arabinogalactan degradation	PF07745 Glycosyl hydrolase family 53	349.30	[91]	GROS_g08150
	PL3 Pecate lyase	Pectin degradation	PF03211 Pectate lyase	137.06	[92, 93]	GROS_g04366
						GROS_g05398
						GROS_g07968
Plant defense manipulation	GH18 chitinase	Chitin degradation	PF00704 Glycosyl hydrolase family 18	2.30	[94]	GROS_g11136
	Chorismate mutase	Conversion of Chorismate into SA	PF01817 Chorismate mutase type II	42.36	[95]	GROS_g02441
						GROS_g08190
	Candidate Isochorismatase	Conversion of Chorismate into SA	PF00857 Isochorismatase family	66.08	[96]	GROS_g01640
Detoxification	Candidate Cyanate lyase		PF02560 Cyanate lyase C-terminal domain	11.51	[17, 24]	GROS_g09531
Nutrient processing	GH32 invertase	Degradation of sucrose in glucose and fructose	PF00251 Glycosyl hydrolases family 32 N-terminal domain	241.26	[22, 23]	GROS_g05724
						GROS_g06434
						GROS_g08674
						GROS_g09735
						GROS_g09969
						GROS_g10583
						GROS_g11374
						GROS_g11397
						GROS_g11793
						GROS_g13274
						GROS_g14232
	VB1 thiD	Vitamin B1 biosynthesis	PF08543 Phosphomethylpyrimidine kinase	154.50	[97]	GROS_g07352
	VB1 thiE	Vitamin B1 biosynthesis	PF02581 Thiamine monophosphate synthase/TENI	163.99	[97]	GROS_g07353
	VB1 thi4	Vitamin B1 biosynthesis	PF01946 Thi4 family	108.07	[97]	GROS_g10855
	VB1 thiM	Vitamin B1 salvage	PF02110	46.05	[97]	GROS_g07354
			Hydroxyethylthiazole kinase family			
	VB1 tenA	Vitamin B1 salvage	PF03070	108.33	[97]	GROS_g05327
			TENA/THI-4/PQQC family			GROS_g07355
	VB5 panC	Vitamin B5 biosynthesis	PF02569	183.11	[97]	GROS_g05752
			Pantoate-beta-alanine ligase			
	VB6 aSNO	Vitamin B6 biosynthesis	PF01680	12.72	[98]	GROS_g08956
			SOR/SNZ family			
	Candidate PolS Polyglutamate synthase	Not known	PF09587	102.00	[99]	GROS_g07961
			Bacterial capsule synthesis protein PGA_cap			
	Candidate GSI Glutamine synthase	Nitrogen assimilation	PF00120	29.24	[100, 101]	GROS_g02362
			Glutamine synthetase, catalytic domain			
Feeding site induction	NodL - like	Candidate acetyltransferase	PF12464 Maltose acetyltransferase	13.12	[100, 102]	GROS_g11033
			PF00132 Bacterial transferase hexapeptide (six repeats)			
Not known	Candidate L-threonine aldolase	??	PF01212 Beta-eliminating lyase	164.69	[100]	GROS_g10421
						GROS_g10422
						GROS_g10423
	Candidate Phosphorybosyl transferase	??	PF00156 Phosphoribosyl transferase domain	198.13	[100, 101]	GROS_g04632
						GROS_g06735

**Fig. 4:**
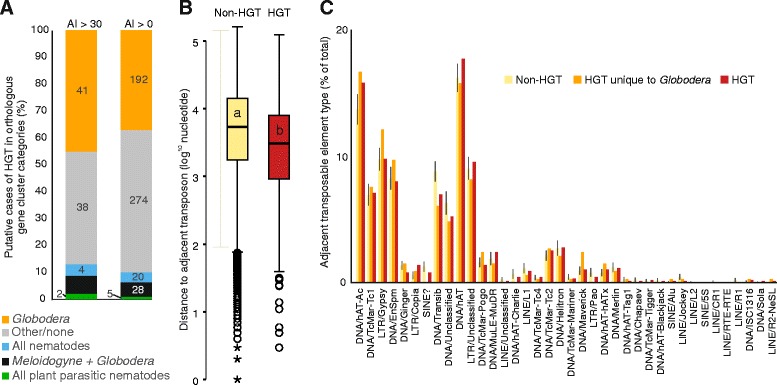
Analysis of genes putatively acquired by HGT. **a** Using an AI >30 or >0, between 45 % and 36 % of putative HGT genes are present in orthologous gene cluster categories unique to the *Globodera* and may give an insight into the relatively recent HGT history since the *Globodera*–*Meloidogyne* divergence. **b** Genes putatively acquired by HGT (AI >0) are significantly closer to transposable elements when compared to all other genes not predicted to be acquired by HGT (*p* <0.000, Mann–Whitney U test). **c** There was no significant association of any independent class of transposable element with genes putatively acquired by HGT. Despite the divergent transposable element composition of *Globodera* (Additional file 11: Figure S6), these were not associated with putative HGT events specific to *Globodera*

Protein domains were identified in 65 % of the putative HGT proteins with an AI >0 and 88 % of those with an AI >30 (Additional file 10: Table S3). The HGT candidates included a set, with AI >29, with predicted functions in plant cell wall modification and degradation, including GH5 cellulases, expansin-like proteins, GH53 candidate arabinogalactan endo-1,4-beta-galactosidase and PL3 pectate lyases. Other cases of HGT may be involved in nutrient processing. A GH32 protein from *G. pallida* has been shown to be a functional invertase expressed in the digestive system [34]. This enzyme may convert sucrose, the main circulating form of sugar in plants, into glucose and fructose which are readily usable by nematodes. We identified 11 GH32-bearing proteins in *G. rostochiensis*, suggesting that this function may be especially important. The phylogenetically dynamic pattern of HGT into tylenchomorph genomes is illustrated by the absence of GH30_8 xylanases, GH28 polygalacturonase as well as GH43 candidate arabinanase in *G. rostochiensis* and *G. pallida*, despite their presence in root-knot nematodes [16]. Furthermore, of the 91 genes with AI >30, six are present in orthologous protein clusters unique to the *Globodera* and *Meloidogyne*, yet many classes are functionally represented in both genera, consistent with multiple acquisitions. The distribution of putative HGT cases across orthologous gene categories is broadly consistent between AI >0 and AI >30. Both suggest a substantial proportion of genes putatively acquired by HGT (36–45 %) are unique to the *Globodera* and may give an insight into the relatively recent HGT history since the *Globodera–Meloidogyne* divergence (Fig. 4a). Three-quarters of genes with AI >30 and unique to *Globodera* are present in differential expression super-clusters, the most common of which are super-clusters 13 and 20 which, respectively, describe genes specifically upregulated during infective J2 and parasitic females. This may indicate that these genes (several candidate invertases, candidate L-threonine aldolase and VB1 tenA (Additional file 10: Table S3)) are functionally deployed during parasitism following horizontal transfer. Although transposable elements (TEs) are closely associated with putative HGT events (*p* <0.001, Mann–Whitney U test, Fig. 4b), the divergent transposable element assemblage in *Globodera* species compared to other tylenchomorphs (LINE/Jockey and SINE/Alu, Additional file 11: Figure S6) is not preferentially associated with putative HGT cases also specific to the *Globodera* (Fig. 4c).

### Effectors in *G. rostochiensis* are sequence diverse between pathotypes

Effectors play central roles in both pathogenicity and virulence. The evolution of virulence on a particular host or variety can involve both gain and loss of effector function. Effectors may become specialised to function in a new host [35], while effector gene loss (or loss of expression) may allow a pathogen to evade recognition [36]. We identified *G. rostochiensis* effectors by sequence similarity to effectors with experimentally verified gland cell expression in related taxa (*Heterodera*, *Globodera*). Many effectors in plant-parasitic nematodes are members of large multi-gene families, only a subset of which are effectors [10, 13, 37]. For example, in *G. pallida* there are ~300 SPRY (PF00622) domain containing proteins, fewer than 10 % of which are deployed as effectors [13]. We therefore further filtered the potential effector set for the presence of a signal peptide for secretion and absence of a transmembrane domain to retain a high confidence list of 138 loci (Additional file 12: Table S4), including 101 genes similar to sequences expressed in the dorsal gland cell, 35 genes similar to those expressed in subventral gland cells and two genes similar to those expressed in the amphid sheath cells. The set included representatives of 37 different effector gene families (Additional file 12: Table S4). The vast majority of these effectors (116/138) exhibited expression profiles consistent with a role in parasitism (Additional file 12: Table S4), as would be expected for effectors. The temporal expression profiles of dorsal and subventral effectors were also consistent with the observed changes in activity of these glands throughout nematode development [38–41]. Most subventral gland effectors were primarily expressed at J2, while dorsal gland effectors were expressed at J2 and/or 14 dpi. Approximately 8.5 % of genes putatively acquired via HGT (8.47 % of those with AI >0 and 8.79 % of those with >30) are present on the stringent effector list; examples of which include putative pectate lyase, beta - 1,4 - endoglucanase and expansins.

Intra-species variation within the *G. rostochiensis* effectorome was examined by mapping whole genome resequencing data from nine populations across five pathotypes (Ro1, Ro2, Ro3, Ro4 and Ro5) to the reference assembly (pathotype Ro1). A total of 1,081,802 variants were detected, of which 794,505 were single nucleotide polymorphisms (SNPs) and 283,434 were insertions/deletions (indels) (Additional file 13: Table S5). Homozygous molecular markers descriptive of pathotypes 4 and 5 were identified (Additional file 14: Table S6). Interestingly, no variants were descriptive of all Ro1, Ro2 or Ro3 populations. Consistent with this, a maximum likelihood phylogeny constructed from 730,705 genome wide SNPs identifies two distinct groups of Ro1, together separate from Ro2, Ro3, Ro4 and Ro5 (Additional file 15: Figure S7A). The variation within pathotype Ro1 is as great as, if not greater than, the variation between Ro1 and the other pathotypes (Additional file 15: Figure S7B).

A total of 108 *G. rostochiensis* effectors (78 %) contained predicted modification of function (non-synonymous mutation) and/or predicted loss of function (frame shift indel or premature stop codon) in at least one pathotype. When accounting for gene length, *G. rostochiensis* effectors did not show significantly different numbers of predicted loss of function variants, but did contain significantly more total variants and more predicted modification of function variants per gene (*n* = 131, Mann–Whitney U test, *p* <0.028 and *p* = 0.003, respectively), compared to randomly selected non-effector genes. No individual variant was homozygous for the reference allele in all populations avirulent on H1 (Ro1 and Ro4) and homozygous for the variant allele in all populations virulent on H1 (Ro2, Ro3 and Ro5). This observation is consistent with the suggestion that distinct populations of Ro1 (Additional file 15: Figure S7 and [42]), in addition to Ro4, have evolved the same phenotype on H1 independently [8]. Convergent evolution of the same phenotype by independent mutations may be explained by identifying genes which are always homozygous present for at least one predicted loss or change of function variant in populations virulent on H1 and always homozygous absent for any predicted loss or change of function variants in populations avirulent on H1. No such cases were identified from these population sequencing data. However, 190 genes were identified with at least one predicted modification or loss of function variant homozygous absent in all avirulent populations and homozygous or heterozygous present in virulent populations. When cross-referenced with the high-confidence effector list, this was reduced to two genes. Gene g13394 is similar to GLAND10 [43], which encodes a putative cellulose binding protein and originates from the subventral gland cell. Gene g12477 is similar to the 3H07_Ubiquitin_extension effectors that are expressed in the dorsal gland cell [44, 45], and are involved in host immune suppression [46]. Forty-eight SNPs were identified in 19 non-effector genes with a difference in average allele frequencies of 70 % or higher between virulent and avirulent populations and a minimal difference in allele frequencies of 25 % between individual virulent and avirulent populations (Additional file 16: Table S7), of which four SNPs were located in g03129, a Ryanodine receptor-like containing three SPRY domains, and seven in g09064, a molecular chaperone from the Hsp90 family.

### Effectors in the *G. rostochiensis* genome are compartmentalised into islands

**Fig. 5:**
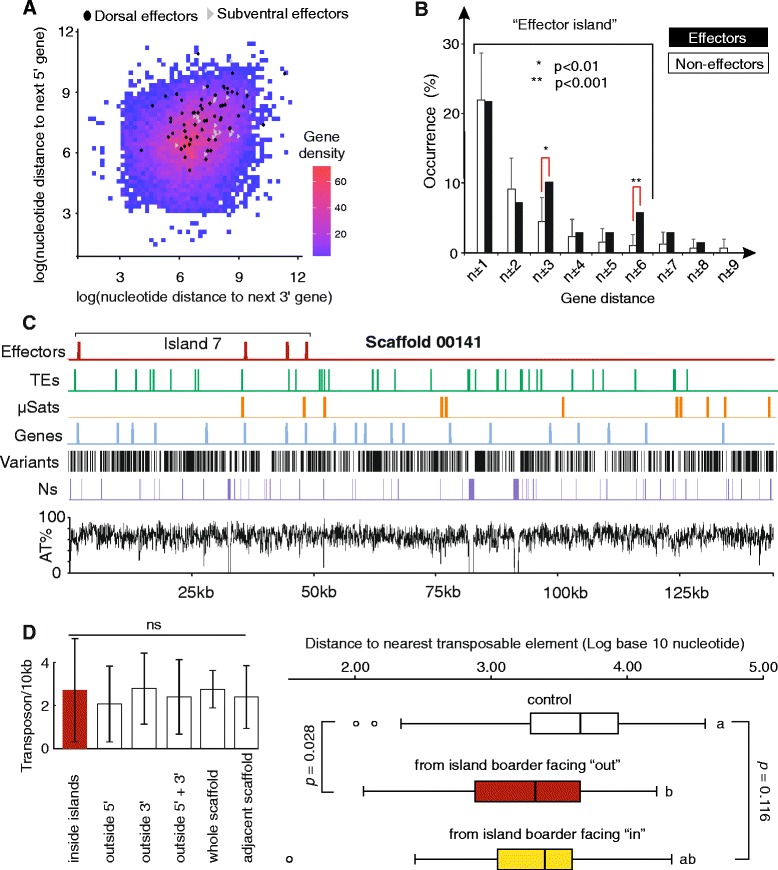
Effectors in *G. rostochiensis* are grouped into ‘islands’. **a** Dorsal (*black*) or subventral (*grey*) effectors are skewed towards a higher neighbouring gene distance compared to random (Student’s t-test, *p* <0.01), yet are contained within gene dense regions of the genome. **b** The presence of effectors in adjacent (n ± 1), or neighbouring positions (up to ±9), was determined. As a negative control, a subset of 612 *G. rostochiensis* gene families not predicted to contain effectors was identified from the OrthoMCL. Starting from this initial negative set of 612 gene families, 37 of these gene families were selected at random and the presence of genes from these 37 families in adjacent (n ± 1), or neighbouring positions (up to ±9), was determined. This process was repeated for 1000 iterations to generate a robust negative for the average frequency in each neighbouring position. The observed frequency of effector occurrence at each position (*black bars*) was compared to the average of 1000 iterations for the negative (*white bars*). For non-effector containing gene families, increasing distance from each gene reduces the likelihood of identifying another member in any of the same families (error bars indicate standard deviation of 1000 iterations). The clustering of effector loci extends beyond immediate neighbours, with an excess of effector loci as next-but-two neighbours (*n* ± 3) and also at n ± 6 (χ^2^, *p* 0.01 and 0.001, respectively). **c** Example of one island (7) at the edge of scaffold 00141. With the exception of high effector density (*red*), no obvious genetic features are associated (gaps (Ns, *purple*), AT content (*black line*), gene density (*blue*) microsatellites (*orange*), variants (*black bars*) and transposable elements (TEs, *green*)). **d** No difference in transposon density was found between islands, in the remainder of scaffolds containing islands, in entire scaffolds containing islands or in scaffolds numerically adjacent to those containing islands (Kruskal–Wallis, *p* = 0.515, error bards indicate standard deviation). When each island is treated as a single locus, the nearest external transposable element 5′ of the first gene, and 3′ of the last, is significantly closer than expected (ANOVA, n = 39, *p* = 0.028 accounting for multiple testing, Fig. 5d). Interestingly, the inverse measurement (the closest internal transposon to each island border), is not significantly closer than expected (*n* = 45, *p* = 0.116, Fig. 5d), suggesting that this may be a feature of islands as an integral whole, rather than the separate genes comprising the islands

In several unrelated eukaryotic plant pathogens, effectors are not randomly distributed in the genome, but are rather located in specialised regions. For example, in *Phytophthora infestans* most effectors are located in gene-sparse regions of the genome and it is proposed that this facilitates rapid evolution and adaptation [47]. Comparatively, *G. rostochiensis* effectors were located in gene-dense regions of the genome (Fig. 5a), albeit with a skewed distribution of gene density compared to an identically sized subset of non-effectors (Student’s *t*-test, *n* = 138, *p* <0.001, Additional file 17: Figure S8). Compared to an expectation of 2 % for a random set of 138 genes, the 138 high-confidence effectors had another high-confidence effector as an immediate chromosome neighbour in 22 % of cases (*χ*^2^, *p* <0.0005). This excess was due to local tandem duplication, as effectors that were directly adjacent to one another in the genome were often from the same effector family, and were frequently more similar to the adjacent gene than to other members of the same gene family located elsewhere in the genome. Such local tandem duplication is a common feature of gene families in *G. rostochiensis* (Fig. 5), however, groups of functionally related gene families (i.e. effectors) tend to be in clustered in genomic islands. For a random subset of 37 non-effector containing gene families, increasing distance from each gene reduces the likelihood of identifying another member in any of the same 37 families. However, the clustering of effector loci extends beyond immediate neighbours, with an excess of effector loci as next-but-two neighbours (n ± 3) and also at n ± 6 (*χ*^2^, *p* <0.01 and 0.001, respectively, Fig. 5b). Over one-third of all effectors were described by 21 effector islands of 2–4 effector loci (Additional file 18: Table S8) with an average length of ~20 kb. Over half of the islands included effectors from more than one effector gene family, yet 80 % comprised genes expressed in only one cell type (dorsal gland cell, subventral gland cell). Several islands included loci similar to other effectors not included in the high-confidence list.

**Fig. 6:**
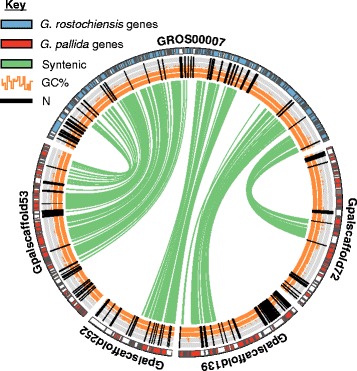
Synteny between *G. rostochiensis* and *G. pallida. G. rostochiensis* genes (*blue*) in scaffold7 (500 kb) are syntenic (*green arcs*) with *G. pallida* genes (*red*) on four scaffolds. Synteny breakpoints primarily co-occur with large insertions in the *G. pallida* assembly. GC content and regions of undetermined sequence are represented by *orange* and *black bars*, respectively

*G. rostochiensis* effector islands were also identified in *G. pallida*. Effector islands containing more than one one-to-one orthologue were similarly arranged in close proximity in *G. rostochiensis* and *G. pallida*, with just three exceptions. One island in *G. rostochiensis* was split across the ends of two scaffolds in *G. pallida*, suggesting the split in *G. pallida* may be an artefact of gapped assembly. Two other *G. rostochiensis* islands were dispersed in *G. pallida*, across different large scaffolds. Synteny between the genome assemblies of *G. rostochiensis* and *G. pallida* extends beyond effector islands, despite the fragmented nature of both assemblies. Based on OrthoMCL protein cluster-membership, 109 distinct syntenic clusters of scaffolds which contained runs of at least five syntenic proteins each were identified, involving 249 *G. pallida* and 202 *G. rostochiensis* scaffolds (Additional file 19: Figure S9). In total, 38.2 Mb of *G. pallida* (36.9 % of the genome) scaffolds are partially syntenic to 31.1 Mb (34.0 % of the genome) of *G. rostochiensis* scaffolds (ignoring N’s). Breakage of synteny between two scaffolds was observed in 20 pairs, seven of which involved inversions. The low proportion of syntenic regions most likely reflects the draft nature of both assemblies (*G. pallida* scaffolds in clusters: 12 % N’s; *G. rostochiensis* scaffolds in clusters: 4.9 % N’s). A subset of the largest syntenic cluster is shown in Fig. 6. Synteny breakpoints which primarily co-occur with large insertions in the *G. pallida* assembly may suggest that large-scale rearrangements have taken place during their divergence and yet effector islands remain predominantly intact. Long-range DNA-sequencing data will prove crucial for assessing the true proportion of syntenic scaffolds and estimating the amount of synteny breakage.

Identifying features enriched within effector islands in *G. rostochiensis* remains challenging; there is no evidence for more AT-rich sequences, contig break points, polymorphisms or microsatellite repeats within islands, flanking islands or scaffolds containing islands (Fig. 5c). However, despite no difference in transposon density within islands (2.7/10 kb ±2.4), in the remainder of scaffolds containing islands (2.4/10 kb ±1.7), in entire scaffolds containing islands (2.8/10 kb ±0.9) or in scaffolds numerically adjacent to those containing islands (2.4/10 kb ±1.5, Kruskal–Wallis, *p* = 0.515, Fig. 5d), transposable elements are closely associated to island borders. When each island is treated as a single locus, the nearest external transposable element 5′ of the first gene, and 3′ of the last, is significantly closer than expected (ANOVA, *n* = 39, *p* = 0.028 accounting for multiple testing, Fig. 5d). Interestingly, the inverse measurement (the closest internal transposon to each island border), is not significantly closer than expected (n = 45, *p* = 0.116, Fig. 5d), suggesting that this may be a feature of islands as an integral whole, rather than the separate genes comprising the islands.

### Identification of a putative enhancer motif associated with dorsal gland effectors

**Fig. 7:**
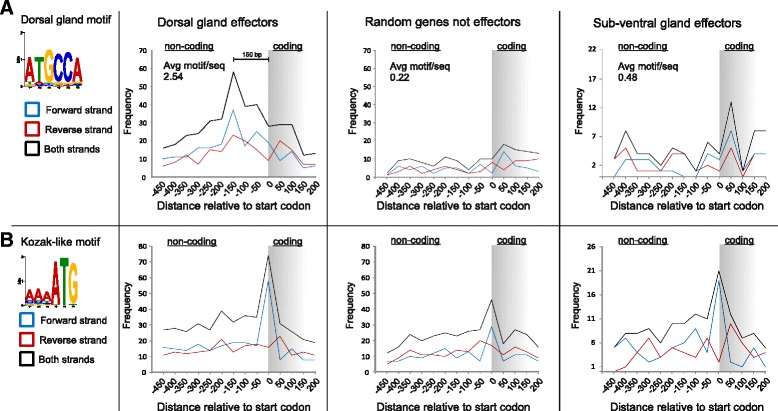
DOG box: a six-nucleotide motif enriched in the promoter region of dorsal gland effectors. **a** The ATGCCA motif is specifically enriched in the promoter region of dorsal gland effectors compared to non-effectors and subventral gland effectors. On average, DOG box-containing dorsal gland effectors contain ~2.54 copies of the motif in their promoter region, compared to ~0.22 for an identical sized random subset of non-effectors or ~0.48 for subventral gland effectors. The frequency of this motif peaks 150 bp upstream of the start codon and is not strand-specific. **b** A strand-specific, Kozak-like motif, which includes the start codon (AAAATG), can be seen for dorsal, subventral and non-effectors at the predicted start of the coding sequence, indicating that predictions of translation start sites are accurate. No substantial cross-identification between each motif is seen

The existing roster of effector proteins in plant-parasitic nematodes has been defined through painstaking and exacting experimental studies employing gland cell-specific complementary DNA (cDNA) sequencing and in situ hybridisation [43]. We therefore sought possible regulatory motifs associated with the highly tissue specific expression pattern of effector genes that might act as an alternative criterion for their identification *in silico* [10, 48]. By employing a differential motif discovery algorithm which normalises for GC content (HOMER) [49], we identified a short DNA motif (the DOrsal Gland motif or DOG box, ATGCCA), specifically enriched in the promoter region (500 bp upstream of the start codon) of genes sequence-similar to experimentally validated dorsal gland cell effectors, compared to either sub-ventral gland effectors or all other non-effectors (*p* = 1e^–10^). Of the 101 *G. rostochiensis* dorsal gland effectors, 77 % had at least one DOG box in their promoter region. This encompasses 26 of the 28 dorsal gland effector families (92 %) including genes that are unrelated in sequence and ontogeny, yet only 5/10 non-dorsal gland effectors (subventral and amphid). Dorsal gland effectors contained an average of 2.54 DOG boxes in their promoter regions, compared to 0.22 for an identically sized subset of non-effectors, 0.32 for all non-effectors or 0.48 for effectors secreted from subventral glands (Fig. 7a). Motif occurrence peaked 150 bp upstream of the start codon and was not strand-specific. Despite the presence of an ATG within the DOG box, the motif does not arise from specifically mis-predicting the start codon of effectors. A strand-specific, Kozak-like motif peak which includes the start codon (AAAATG) was observed in dorsal, subventral and non-effectors at the predicted start of the coding sequence (Fig. 7b). We were unable to identify a motif that correlated with time of expression (e.g. when comparing dorsal gland effectors expressed at 14 dpi versus dorsal gland effectors expressed at J2) or with expression in the subventral gland. We found no enrichment of DOG boxes upstream of the first gene in tandem series of adjacent dorsal gland cell-expressed effectors arranged in an island.

### The DOG box as a predictor of effectors

**Fig. 8:**
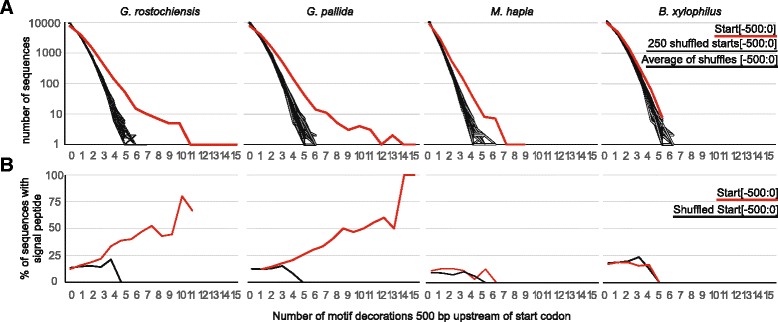
Scope for utility of the DOG box to predict secretory proteins. **a** The number of promoter regions with multiple copies of DOG motifs is higher than random for *G. rostochiensis*, *G. pallida* and *M. hapla*, but not for *B. xylophilus*. Normal promoter regions are shown in *red*, 250 iterations of randomising the sequence of each promoter region are shown in *grey*, the average of which is shown in *black*. **b** For *G. rostochiensis* and *G. pallida*, the more motifs present in the promoter region, the more likely it is that the corresponding gene will contain a signal peptide for secretion (*red line*). Randomising each promoter region abolishes this effect (*black line*). For *M. hapla* and *B. xylophilus*, an increased number of motifs in the promoter regions does not correlate with a greater chance of the corresponding gene containing a predicted signal peptide

We screened the regions 500 bp upstream of all loci in the *G. rostochiensis* genome for DOG boxes on either strand. The number of genes associated with multiple DOG boxes was significantly higher than expected by chance (Fig. 8a). For some genes, nearly one-fifth of the entire 500 bp promoter region comprised ATGCCA motifs. Sequences with more DOG boxes in their promoter regions were more likely to have predicted a signal peptide for secretion (Fig. 8b). These findings suggest that the DOG box may be a strong predictor of secretion, and thus likely effector function, of *G. rostochiensis* genes. The same DOG box motif was also present at a significantly higher frequency than would be expected by chance and was preferentially associated with secreted proteins in *G. pallida* (Fig. 8a, b). In the more distantly related *M. hapla*, the number of genes with multiple occurrences of the motif in their promoter region is higher than expected by chance, but the presence of motifs was not associated with the downstream gene encoding a predicted signal peptide. No enrichment of the DOG box or association with secreted proteins was observed for the much more distantly related *B. xylophilus*. This suggests that in addition to minimal overlap between effector repertoires [22, 50], the control of effector expression in the dorsal gland cell may also require a different motif/s in these nematodes.

Although not all secreted proteins are effectors, all effector proteins are secreted. Within the 150 *G. rostochiensis* genes with three or more DOG boxes and a signal peptide, there were 31 known effectors from 14 families, an approximately 100-fold enrichment. The expression patterns of these 150 genes (including newly discovered candidate effector sequences) were consistent with a role in parasitism. For *G. pallida*, where more comprehensive life stage expression data are available, the same association was observed (Additional file 20: Figure S10) [22]. Despite the fact that most genes with >3 ATGCCA motifs in *G. pallida* and a signal peptide are expressed at J2, the number of motifs in the promoter region was not a quantitative predictor of gene expression at J2 (R^2^ = 0.0002, Additional file 20: Figure S10) or at any other life stage, indicating that the ATGCCA motif is not a J2 enhancer. These data most likely reflect the biology of the nematode which dictates that a substantial proportion of effectors are required in the dorsal gland during the infective juvenile stages.

**Fig. 9:**
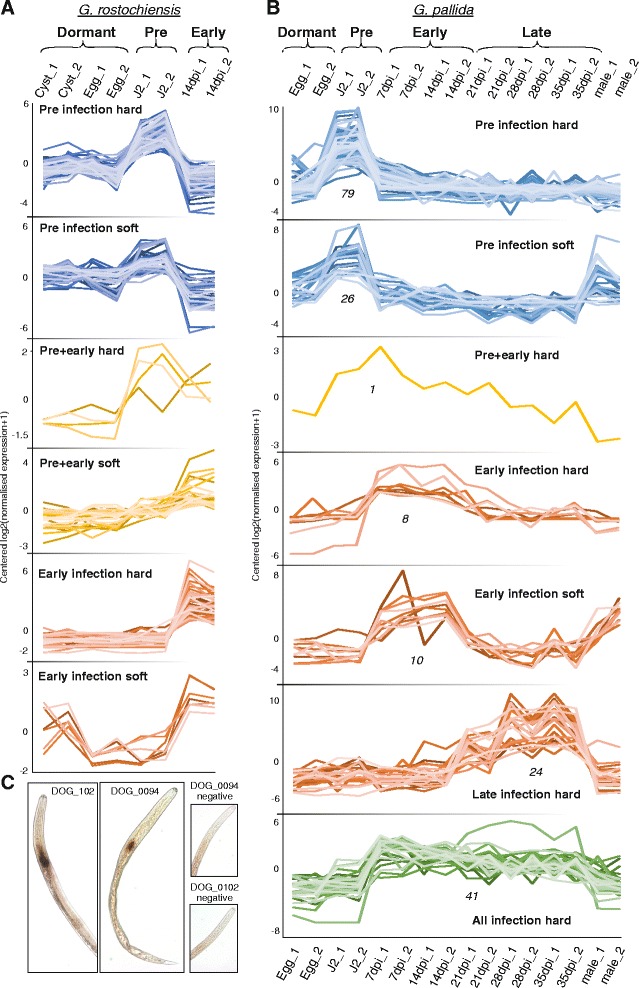
All DOG effectors from *G. rostochiensis* and *G. pallida*. Using a minimum of two DOG boxes, presence of a signal peptide, absence of transmembrane domains and temporal expression profiles consistent with a role in parasitism as selection criteria, we identify and separate all DOG effectors from *G. rostochiensis* (**a**) and *G. pallida* (**b**) into putative functional groups. For both (**a**) and (**b**), clusters were manually assigned to ‘strict’ or ‘inclusive’ subsets of the same overall expression pattern, based on how they conform to the observed pattern. **c** Experimental validation of two novel *G. rostochiensis* DOG effectors confirms the dorsal gland cell expression prediction. DOG_0102 (g04707) contains five DOG boxes in its promoter while DOG_0094 (g14226) contains six boxes

We used an extended set of criteria to predict potential DOG effectors from *G. rostochiensis* and *G. pallida*. Genes with two or more DOG box motifs within 500 bp upstream of the start codon, a signal peptide and no transmembrane domain on the corresponding protein, and temporal expression profiles consistent with a role in parasitism (Fig. 9a, b, Additional file 21: Table S9 and Additional file 22: Table S10) were classified as likely effectors. To validate these criteria, we examined the spatial expression pattern using in situ hybridisation of two new predictions that had no similarity to any published effector. Both exhibited expression in the dorsal gland cell (Fig. 9c), confirming that the DOG box, in combination with other criteria, can act as a predictor of novel effector candidates. Novel gland cell protein g14226 was clustered in a genomic island with several other similar genes with multiple DOG boxes in their promoter region, another signature of canonical *Globodera* effectors. As biological understanding of dorsal (and other) gland expression in tylenchid plant parasites grows, it may be possible to refine the interpretation of DOG box presence and clustering and also develop understanding of the control of gland cell expression of effectors in other taxa.

## Conclusions

The interactions between plant-parasitic nematodes and their hosts are both complex and specific. In a successful interaction, the nematodes can avoid induction of an effective host immune response, resist any immune response that is expressed and manipulate the host’s developmental and cell biology to induce and maintain a functional feeding site. These interactions are mediated by an armoury of effectors that plant-parasitic nematodes appear to have assembled from adaptation of endogenous genes and also loci acquired by horizontal gene transfer from a diverse range of other taxa. To probe and understand these interactions, genomic analyses complement more directed studies, to drive and focus future programmes. Genomics can deliver whole-system analyses that permit global recovery of likely actors in parasite-host interactions. In turn, these insights can suggest new approaches to the understanding of pathogenesis and ultimately control of parasite-induced crop losses. The expanded effector set, including new effector types, the association of presence of particular effector loci with breaking of plant resistance and the definition of shared transcriptional control systems we have reported here from genomic and transcriptomic analyses of *G. rostochiensis* are demonstrations of this utility.

## Methods

### Nematode culture and DNA isolation

*G. rostochiensis* populations Ro1, Ro2, Ro3, Ro4 and Ro5 from the JHI PCN collection were maintained on a mixture of susceptible varieties in glasshouse conditions. For the reference assembly (Ro1), DNA was extracted according to described methods [22]. For population re-sequencing, DNA extraction was carried out as previously described [42].

### Genome sequencing and assembly

Three sequencing libraries were prepared from total genomic DNA (Additional file 23: Table S11). A PCR-free 400–550 bp paired-end Illumina library was prepared using a previously described protocol [51], with the addition of sample clean up and size selection with Agencourt AMPure XP. DNA was precipitated onto beads after each enzymatic stage with an equal volume of 20 % Polyethylene Glycol 6000 and 2.5 M sodium chloride solution. Beads were not separated from the sample throughout the process until after the adapter ligation stage: fresh beads were then used for size selection. Two mate pair libraries with ~2 kb virtual insert size were constructed [52]. The libraries were denatured using 0.1 M sodium hydroxide and diluted to 8 pM in hybridisation buffer for cluster amplification on the Illumina cBOT using the V3 cluster generation kit following the manufacturer’s protocol, followed by a SYBRGreen cluster density QC prior to paired-end 100 base sequencing on an Illumina HiSeq2000. Raw data were analysed using the Illumina RTA1.8 analysis pipelines.

An initial assembly was produced from a combination of short-fragment paired-end and mate-pair Illumina libraries (Additional file 23: Table S11). Short paired-end sequence reads were first corrected and initially assembled using SGA v0.9.7 30 [53]. This draft assembly was then used to calculate the distribution of k-mers for all odd values of k between 41 and 81, using GenomeTools v.1.3.7 [54]. The k-mer length for which the maximum number of unique k-mers were present in the SGA assembly (k = 63) was then used as the k-mer setting for de Bruijn graph construction in a second assembly with Velvet v1.2.03 32 [55]. The mate-pair library was then used to further scaffold this Velvet assembly using SSPACE [56] with an iterative approach, in which the number of read-pair links required to scaffold two contigs was initially set to 50, then reduced to 30, 20 and finally set to 10 for two final iterations of SSPACE to produce assembly nGr.v0.9. The three whole genome sequencing libraries were subsequently used to gap fill the assembly (GapFiller v1.10 [57], 10 iterations and default values for extension parameters), producing the final assembly nGr.v1.0.

A BlobDB (Blobtools v0.9.9 (https://drl.github.io/blobtools/) [58, 59] was constructed using: (1) the assembly; (2) similarity search results against the NCBI Nucleotide database (BLASTn 2.3.0+ [60] megablast, E-value cutoff 1e^–65^), Uniref90 (Diamond v0.7.12 [61], blastx, using the options --sensitive, -k 25 and -c 1) and the *G. pallida* reference genome nGp.v1.0 (BLASTn megablast, E-value cutoff 1e^–65^); (3) the three DNA-seq read libraries mapped back to the assembly (CLC mapper v4.21.104315 CLCBio, Copenhagen, Denmark). A Taxon-Annotated-Gc-Coverage plot (TAGC) was drawn at the rank of phylum and under taxrule ‘bestsum’. Using Blobtools view, taxonomically annotated non-nematode scaffolds with a bit-score ≥200 were inspected manually and compared against NCBI Nucleotide database (BLASTn). Twenty-three scaffolds could be excluded as contaminants based on strong similarity to Bacteria or Fungi (span = 98.2 kb). TAGC plots pre- and post-filtering are shown in Additional file 24: Figure S11. SSU/LSU rDNA screening was carried out through sequence similarity searches (BLASTn megablast) of the assembly against SILVA SSUParc and LSUParc databases. Hits were only observed against *G. rostochiensis* SSU (scaffolds GROS_00919, GROS_01231) and LSU (scaffold GROS_00803, GROS_00919, GROS_01231).

### Genome annotation

Genome annotation was carried out in a two-step process detailed in the Additional file 2: Supplementary information. An initial round of automated gene predictions (nGr.v0.9.auto, 13,650 models) were refined in the collaborative genome annotation editor WebApollo (v1.0.4-RC3 [62]). Approximately one-eighth of the gene models were manually inspected based on homology to known *Globodera* genes, RNA-seq evidence and WGS read coverage yielding 1566 manually curated gene models (nGr.v0.9.manual). A second round of de novo gene prediction was carried out on assembly nGr.v1.0 with the addition of manual annotations as protein homology evidence and mapped RNA-seq reads as intron-hints to train and run Augustus (v3.1 [63]) resulting in the final gene set nGr.v1.0 containing 14,309 protein-coding genes. Functional annotation was performed using InterProScan5 (v5.7-48.0 [64]) to identify motifs and domains in the proteins by comparing them against databases Gene3D, PRINTS, Pfam, Phobius, ProSitePatterns, ProSiteProfiles, SMART, SUPERFAMILY, SignalP_EUK, TIGRFAM, TMHMM, Annot8r with KEGG, GO, EC, tRNAscan and rfam. GO-Term annotation and GO-enrichment analysis was carried out using Blast2GO 3.1.3 [65].

### Splicing

Splice sites were extracted from the genomes and GFF3 files present on WormBase for the species in Additional file 4: Figure S2, using custom script extractRegionFromCoordinates.py (https://github.com/DRL/GenomeBiology2016_globodera_rostochiensis/GNU GENERAL PUBLIC LICENSE). Four base pairs up and downstream of the 5′ donor site, and 6 bp upstream of the 3′ acceptor site were used to construct a consensus sequence for all GC/AG introns, and an identical sized sample of randomly selected GT/AG introns, using MEME SUITE v4.9.1 [66].

### Transcriptome sequencing and differential expression

RNA from two life stages (hatched second-stage juvenile and 14 dpi female) was sequenced, each in biological duplicate, with Illumina Hiseq 100 bp paired-end reads (SRA accessions ERR202479, ERR202487 and PRJEB12075). These were compared with two additional life stages (dormant cysts and hydrated eggs), similarly sequenced in biological duplicate (Genbank accessions SAMN03393004 and SAMN03393005). All RNA-seq was carried out on pathotype Ro1. Normalized gene expression values and differentially expressed genes were identified as previously described [50]. In brief, raw reads were trimmed of adapter sequences and low quality bases (Phred <22, Trimmomatic [67]), mapped to the genome (Tophat2, [68]), counted on a per gene basis (bedtools v2.16.2 [69]), TMM normalised and differential expression analysis and clustering were performed using a Trinity wrapper pipeline and associated scripts for RSEM [70] and EdgeR [71] (FDR <0.001, minimum fold-change 4, [72]). Expression clusters were grouped based on the tree height parameter (12 %) and manually assigned to expression super-clusters.

### Phylogenetic analysis of WAGO proteins

Putative *G. rostochiensis* (n = 23), *G. pallida* (*n* = 18) and *M. hapla* (n = 18) WAGOs present in OrthoMCL clusters, which contained at least one *G. rostochiensis* protein with highest similarity to *C. elegans* WAGO1, were aligned to 545 WAGO sequences from Buck and Blaxter, 2013 [30]. This comprised WAGOs from Clade I, Clade III, Clade IV and Clade V nematodes, as well as non-Nematode argonaute sequences (http://datadryad.org/resource/doi:10.5061/dryad.5qs11). Alignment was carried out using clustal-omega 1.2.0 [73] and alignment was trimmed to only include the core PIWI PAZ domain section of argonautes. The WAG + G + F model of amino acid sequence evolution was selected under AICC using Prottest 3.4 [74] and phylogenetic trees were inferred using RAxML 8.1.20 [75] (ML search + 100 rapid bootstraps).

### Horizontal gene transfer

Candidate horizontal gene transfers (HGT) were detected as previously for plant-parasitic nematodes [37] by calculating AIs as described in [32, 33] using Alienness [31]. Briefly, AIs were calculated for each *G. rostochiensis* protein returning at least one similar sequence in either a metazoan or non-metazoan species (E-value threshold of 1e^–3^) present in NCBI’s non-redundant (nr) database, according to the following formula:

$$ AI = \log \left( best\  metazoan\kern0.37em e\_ value+\kern0.37em {e}^{-200}\right) - \log \left( best\ non\_ metazoan\kern0.37em e\_ value+\kern0.37em {e}^{-200}\right) $$

Sequences derived from species under NCBI Taxonomy’s ‘Tylenchida’ (TaxID: 6300, equivalent to Tylenchomorpha) were not included in this calculation to allow detection of HGT events which took place in an ancestor of cyst nematodes and their tylenchomorph relatives. No AI value could be calculated for proteins returning no similar sequences in the nr database. An AI >0 indicates a better hit to a non-metazoan species than to a metazoan species and thus a possible acquisition via HGT. An AI >30 corresponds to a difference of magnitude e^10^ between the best non-metazoan and best metazoan E-values and is estimated to be a strong indication of a HGT event [32]. Proteins with an AI >0 and ≥70 % identity to a non-metazoan protein were considered putative contaminants and not included in further analysis.

### Effector identification

Genes in the *G. rostochiensis* genome sequence similar to previously reported effectors with experimentally validated gland cell expression were identified in a two-step process. An inclusive list of effectors was generated by sequence similarity alone. For those effectors that are characterised by the presence of particular domains (e.g. the SPRY domain of SPRY-SEC effectors), hmmsearch [76] using the appropriate domain was used to identify all sequences predicted to contain the same domain using the gathering significance threshold. For all other effectors, BLASTp was used to identify similar sequences (E-value ≤1e^–5^). Cell wall degrading enzymes (CWDEs) identified as putatively acquired via HGT were included if they had known in situ localisation to either gland cell. This inclusive list was triaged by removing those without a predicted signal peptide and/or those with one or more transmembrane domain (Phobius [77]), producing the high-confidence effector list (Additional file 12: Table S4).

### Variant analysis

Sequence reads (Bioproject PRJNA305631) were mapped against the assembly using bwa mem v0.7.12-r1044 [78]. Duplicated read pairs were removed using Picard (http://broadinstitute.github.io/picard). Variants were called using freebayes v0.9.20-16-g3e35e72 [79]. Haplotypes and other complex variants were decomposed using vcflib vcfallelicprimitives v1.0.0-rc0 (https://github.com/ekg/vcflib/releases/tag/v1.0.0-rc0) followed by normalisation using vt normalize v0.57 [80]. The resulting VCF file was filtered with the following parameters: DP > 10 & MQM > 30 & QUAL > 1 & QUAL/AO > 10 & SAF > 2 & SAR > 2 & RPR > 1 & RPL > 1 using vcffilter from vcflib. Variants were annotated using SnpEff v4.1 L [81]. The resulting VCF file was analysed using vt peek, RTG Tools [82] and parse_snpeff.py. Variants (vcf file) were filtered to retain only SNPs (TYPE = snp) with no missing data, 730,705 loci were found from whole genome data. Allele frequencies at each locus was computed by dividing the reference allele observation count (RO) by the read depth (DP). In the same manner, allele frequencies for SNPs present in non-coding regions (n = 619,886) were computed. Seqboot module in PHYLIP v3.695 [83] was used to make 100 bootstrapped datasets. Maximum likelihood phylogenetic trees of the nine populations of *G. rostochiensis* were calculated with the Contml module based on genome-wide SNP allele frequencies and a majority rule consensus tree was constructed using Consense. Principal component analysis (PCA) were calculated with the prcomp() function from the stats package in R based on genome-wide allele frequencies at these 730,705 loci.

### Protein clustering

Putative one-to-one orthologues between *G. pallida* and *G. rostochiensis* were identified by the reciprocal best BLAST hit method. Both proteomes were compared against each other using BLASTp (v2.2.30+) and the resulting files were processed using the script rbbh.py (https://github.com/DRL/GenomeBiology2016_globodera_rostochiensis GNU GENERAL PUBLIC LICENSE, E-value ≤1e^–25^ and reciprocal-query coverage >75 %). Protein clustering analysis was performed on the proteomes (retrieved from Wormbase WS248) of *B. xylophilus*, *C. elegans*, *M. hapla*, *M. incognita*, *G. pallida* (retrieved from WormBase ParaSite WBPS2) and *G. rostochiensis* (nGr.v1.0) using OrthoMCL (v2.0.9 [84]) (with an inflation value of 1.5) and following the guidelines specified in [84]. Phylogenetically informative sets of clusters were plotted using UpSetR (Release v1.0.0, https://github.com/hms-dbmi/UpSetR/releases [85]). For each of four orthologous gene cluster categories (all nematodes tested, all plant parasites tested, *Globodera* and *Meloidogyne* and *Globodera* alone), the percentage of genes present in each differential expression super-cluster was determined. This value was normalised by the total number of genes present in each given differential expression super-cluster, to return a relative measure of abundance used in Fig. 3.

### Effector islands, synteny and promoter analyses

The presence of effectors in adjacent (n ± 1), or neighbouring positions (up to ±9), was determined. As a negative control, a subset of 612 *G. rostochiensis* gene families not predicted to contain effectors was identified from the OrthoMCL. This subset contained gene families of various sizes, the distribution of which with respect to gene family size 1, 2 and ≥3 was the same as that of the effectors. Starting from this initial negative set of 612 gene families, 37 were selected at random and the presence of genes from these 37 families in adjacent (n ± 1), or neighbouring positions (up to ±9), was determined. This process was repeated for 1000 iterations to generate a robust negative for the average frequency in each neighbouring position. The observed frequency of effector occurrence at each position was compared to the average of 1000 iterations. Non-overlapping islands, delineated by furthest distance at which statistically significant enrichment was observed (±6, *χ*^2^ goodness of fit, *p* <0.001), were manually identified.

Synteny between scaffolds of *G. pallida* and *G. rostochiensis* was assessed based on OrthoMCL-cluster membership of both sets of proteins using i-adhore-3.0.01 ((https://github.com/widdowquinn/Teaching/tree/master/Comparative_Genomics_and_Visualisation/Part_2/i-ADHore) type = family, tandem_gap = 10, gap_size = 15, max_gaps_in_alignment = 20, cluster_gap = 20, q_value = 0.9, alignment_method = gg2, prob_cutoff = 0.001, multiple_hypothesis_correction = bonferroni, anchor_points = 5). Syntenic blocks were visualised as clusters in a graph using parse_iadhore.py. *G. rostochiensis* scaffold GROS_00007 (a member of the biggest syntenic cluster) was plotted with its homologous *G. pallida* scaffolds using circos 0.67-7, including GC-content and BLASTn results at an E-value cutoff of 1e-65.

To analyse putative enhancer elements, sequences 500 bp upstream of genes of interest (termed the promoter regions) were extracted from the genome using get_upstream_regions.py (https://github.com/peterthorpe5 GNU GENERAL PUBLIC LICENSE). Enrichment of motifs between categories (DG versus all, DG versus SvG, etc.) was calculated using HOMER [49], specifying max length of six nucleotides. Instances of the motif were identified in FASTA sequences of promoter regions using the FIMO web server [86].

### In situ hybridisation

The spatial expression patterns of two predicted *G. rostochiensis* dorsal gland effectors were determined in J2s by *in situ* hybridisation as described previously [87]. Single-stranded digoxygenin-labelled DNA probes were synthesised from amplified cDNA fragments using primers g14226F (5′-CCGTTGAGCCGTCGACTAAT-3′) and g14226R (5′-TTTCCCGACGTCCAGTTGAC-3′) or g04707F (5′-AAGGAGCACCATCGTACCAAG-3′) and g04707R (5′-GTTCTGAGCCTTGTTGAAAG-3′).

### Description of additional data files

The following additional data are available with the online version of this paper. Additional file 7: File S1 contains the data matrix of normalised expression values. Additional file 2: Supplementary information file 1 contains various supplemental methods and results.

## Additional files

Additional file 1: Figure S1. Gene duplication in the *G. rostochiensis* and related genomes. Comparing the identity of each gene to the next most similar gene in the genome gives insights into potential duplication within the genome sequence. In a diploid species, with a good assembly and gene prediction, we expect no overrepresentation of duplicates at any particular divergence, as is seen in the genome of *M. hapla*. The *G. rostochiensis* protein set has a very similar distribution to that of *M. hapla*, but in *G. pallida* there is an overrepresentation of genes that are >97 % identical to each other. As reported previously, the protein set from *M. incognita* has a distinct excess of duplicates at ~96 % identity, thought to derive from a hybrid origin, and subsequent aneuploidy changes, of this species [23, 88]. *G. pallida* is not believed to derive from a hybridisation event [26] and so this is probably a reflection of duplication at the assembly stage (i.e. retention of allelic copies of loci because of the high level of heterozygosity in UK populations). (PDF 1365 kb) Additional file 2: Supplementary methods and results. (DOCX 28 kb) Additional file 3: Table S1. Genome annotation. (XLSX 9 kb) Additional file 4: Figure S2. Phylum wide analysis of GC/AG splice sites in nematodes. The percentage of GC/AG splices sites with associated consensus sequences are shown for 17 species against a schematic phylogeny of the phylum Nematoda (adapted from [89]). *Red numbers* indicate those which likely represent under reporting due to over-strict parameter settings during gene prediction. (PDF 1371 kb) Additional file 5: Figure S3. Comparison of gene expression between parasitic *G. pallida* life stages. The key transitions to be captured in terms of gene expression of all genes, and in particular for effectors, is from outside the plant (J2) compared to inside the plant (sedentary female). There is almost no difference in global gene expression between the early sedentary time points [22]. A. 84 % of the variation in expression at 7 days post infection (dpi) is explained by variation in expression at 14 dpi. B. This correlation is even more profound if the analysis is restricted to only the effectors (89 %). Similar correlations are possible between 14 and 21 dpi, albeit of lesser magnitude but an identical trend (60 % correlation for all genes, and 64 % correlations for specifically effectors). This is not the case, however, when comparing J2 and 7 dpi (44 % for all genes, and zero correlation for all effectors). Fourteen dpi provides an ideal intermediate for the sedentary stages. (PDF 4379 kb) Additional file 6: Figure S4. Differential expression super-clusters. Ninety-four percent of all differentially expressed genes are manually grouped into 25 biologically relevant expression super-clusters. For each super-cluster, individual cluster graphs are shown where for all expression displayed as centred log fold-change is in the order, Cyst, Cyst, Egg, Egg, J2, J2, 14 dpi, 14 dpi. (PDF 1669 kb) Additional file 7: File S1. Clusters and normalised expression tables. (XLSX 956 kb) Additional file 8: Figure S5. Phylogenetic analysis of Worm-specific Argonauts (WAGOs). A. A total of 604 nematode and non-nematode argonaute proteins drawn as an *unrooted phylogram*. It is assumed that each subtree (B–F) is effectively rooted by the other subtrees; however, the extreme divergence between these proteins yields low support for some subtrees. B–F Subtrees containing *G. rostochiensis* WAGOs (GrWAGOs). Branch widths of subtrees are drawn proportional to branch support. *Coloured boxes* indicate membership of taxa to phylogenetic groups (nematode Clade I, Clade III, Clade IV and Clade V, non-nematode taxa) and *coloured stars* indicate clades composed entirely of Heteroderidae-WAGOs (*Globodera* spp., *Meloidogyne* spp., *Heterodera glycines*). *Orange dots* indicate GrWAGOs in differential expression super-clusters 19, 20 or 21. GrWAGOs are placed within Nematode WAGO-subclades ALG1/ALG2 (1), RDE1/ERGO1/PRG1/2 (2), WAGO1/2/4/5 (7), CSR/WAGO-III/WAGO-IV (8) and NRDE/WAGO-10/11 (5), *sensu* Buck and Blaxter, 2013 [30]. As expected, no GrWAGOs were observed in WAGO-subclades SAGO2/PPW, WAGO-III/-V and ALG3/ALG4. Subclades NRDE/WAGO-10/11, WAGO1/2/4/5 and CSR/WAGO-III/WAGO-IV show increased numbers of paralogous expansion of these gene families within Clade IV in general and Heteroderidae in particular. B All *Globodera* spp. and *Meloidogyne* spp. exhibit one ALG1/ALG2 orthologue each, which form a clade. C The RDE1/ERGO1/PRG1/2 subtree contains another Heteroderidae-specific clade; however, one *M. incognita* sequence is sister to a subclade of Clade III taxa. This is surprising since the Clade III parasites *A. suum* and *B. pahangi* are thought to have lost the piRNA pathway [30] and may be an artefact. D The NRDE/WAGO-10/WAGO-11 subtree shows an expansion of paralogous Heteroderidae-WAGOs, with three GrWAGOs expressed at 14 dpi. E The WAGO1/2/4/5 subtree depicts two Heterodera-specific expansions, of which the larger subclade contains four GrWAGOs expressed at 14 dpi. F The CSR/WAGO-III/WAGO-IV subtree contains another two Heteroderidae-specific expansion, of which one also includes a sequence from the Clade V nematode *Dictyocaulus viviparus* (*ochre star*). (PDF 64 kb) Additional file 9: Table S2. Alien indices. (XLSX 81 kb) Additional file 10: Table S3. High confidence effectors. (XLSX 13 kb) Additional file 11: Figure S6. Transposable elements in *G. rostochiensis*, *G. pallida* and *Meloidogyne spp.* Quantities of transposable elements from (A) DNA, (B) LTR, (C) LINE and (D) SINE super-familes. *Globodera* spp. contain notably more Jockey (LINE) and Alu (SINE) than the *Meloidogyne* spp. (PDF 623 kb) Additional file 12: Table S4. Genes acquired via HGT in other cyst and root-knot nematodes also found in the genome of *G. rostochiensis*. (XLSX 21 kb) Additional file 13: Table S5. Variants summary. (XLSX 11 kb) Additional file 14: Table S6. Molecular markers of pathotypes Ro1, 4 and 5. (XLSX 24 kb) Additional file 15: Figure S7. Phylogenetic analysis of *G. rostochiensis* pathotypes. A. A maximum likelihood phylogeny based on 730,705 genome wide SNPs. Two distinct groups of Ro1 are together separated from Ro2, Ro3, Ro4 and Ro5. Node labels indicate bootstrap support values for 100 iterations. B. Principle component analysis (PCA) based on the same dataset suggest that for pathotype Ro1, intra-pathotype variation is similar to inter-pathotype variation. REF Ro1 = Reference strain Ro1 assembly, MAR149 Ro1 = British Columbia, QC Ro1 = Quebec, Ro19 Ro1 = European, NFLD Ro1 = Newfoundland, SCRI_Ro2, SCRI_Ro3, SCRI_Ro4, SCRI_Ro5 are pathotype populations from the James Hutton Institute collection. (PDF 1636 kb) Additional file 16: Table S7. SNP allele frequency correlation with virulence in non-effectors. (XLSX 15 kb) Additional file 17: Figure S8. Comparison of distance to neighbouring gene for effectors and non-effectors. A. The log nucleotide distance to next gene 5′ and 3′ for a random subset of non-effectors (*n* = 138). B. The log nucleotide distance to next gene 5′ and 3′ for high-confidence effectors (n = 138). C. Comparing the distance of each gene to its neighbour either upstream or downstream suggests that despite being located in gene dense regions of the genome, effectors have a skewed distribution of gene density compared to an identically sized subset of non-effectors (lower case letters indicate homogenous subsets, Student’s t-test correcting for multiple comparisons *p* <0.001). (PDF 1393 kb) Additional file 18: Table S8. Effector islands. (XLSX 13 kb) Additional file 19: Figure S9. Synteny clusters. Network representation of i-ADHoRe results. Scaffolds are represented as nodes (*blue* = *G. rostochiensis*, *ochre* = *G. pallida*) whose diameter is scaled by scaffold length and vertices are drawn between them if five or more anchorpoints (i.e. syntenic proteins, numbers on vertices indicate count of anchorpoints) have been found. The biggest cluster (*dashed black line*) is composed of 26 *G. rostochiensis* scaffolds (4.99 Mb) and 35 *G. pallida* scaffolds (5.54 Mb). Part of this cluster (*grey dotted line*) is shown in Fig. 6. (PDF 570 kb) Additional file 20: Figure S10. The DOG box is not a quantitative predictor of temporal expression. A. In *G. pallida*, genes with four or more motifs and a signal peptide are primarily expressed during the infective stage. Each line represents the expression pattern of a different gene. B. The number of motifs does not correlate with gene expression at J2 (*black*, all numbers of motifs; *red*, four or more motifs). (PDF 1707 kb) Additional file 21: Table S9. G. rostochiensis putative DOG effectors. (XLSX 36 kb) Additional file 22: Table S10. G. pallida putative DOG effectors. (XLSX 44 kb) Additional file 23: Table S11.
*G. rostochiensis* genome libraries. (XLSX 9 kb) Additional file 24: Figure S11.
*Blob plots*. A. *Blob plot* of the initial *G. rostochiensis* assembly, displaying some minor contamination from Actinobacteria and Ascomycota. Each scaffold is drawn as a circle based on its GC content (*X-axis*) and log-coverage (*Y-axis*), with a diameter proportional to its length and coloured by its taxonomic annotation at the phylum-level. In the legend, colours of phyla are listed together with scaffold-count, scaffold span and scaffold N50. The *histograms above and to the right* of the main scatter plot sum contig spans for GC proportion bins and log-coverage bins, respectively. B. *Blob plot* of the *G. rostochiensis* assembly after removal of contaminant scaffolds. (PDF 8314 kb) Additional file 25: Table S12. Microsatellite abundance. (XLSX 8 kb)
