# Supplementary figures and images for: The genome of the yellow potato cyst nematode, Globodera rostochiensis, reveals insights into the basis of parasitism and virulence
Source: Genome Biol. 2016 Jun 10;17:124. doi: 10.1186/s13059-016-0985-1 (PMC4901422; doi:10.1186/s13059-016-0985-1)

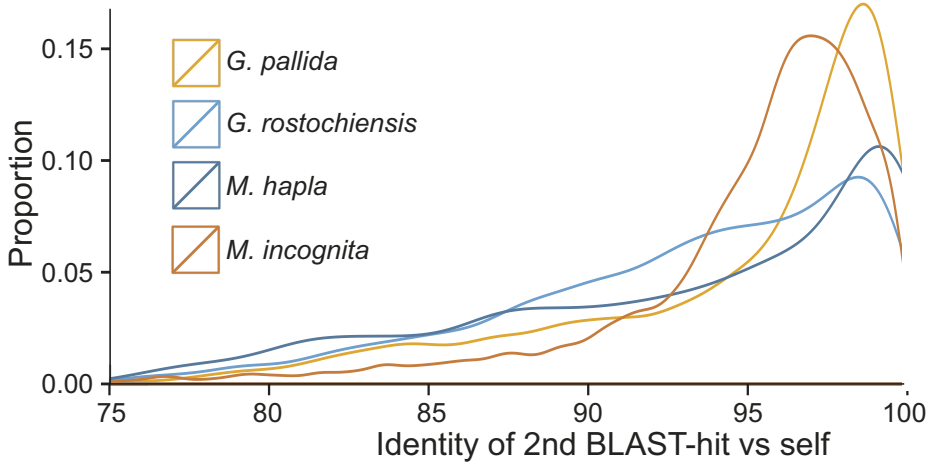

Supplement: Additional file 1: Figure S1. — Gene duplication in the G. rostochiensis and related genomes. Comparing the identity of each gene to the next most similar gene in the genome gives insights into potential duplication within the genome sequence. In a diploid species, with a good assembly and gene prediction, we expect no overrepresentation of duplicates at any particular divergence, as is seen in the genome of M. hapla. The G. rostochiensis protein set has a very similar distribution to that of M. hapla, but in G. pallida there is an overrepresentation of genes that are >97 % identical to each other. As reported previously, the protein set from M. incognita has a distinct excess of duplicates at ~96 % identity, thought to derive from a hybrid origin, and subsequent aneuploidy changes, of this species [23, 88]. G. pallida is not believed to derive from a hybridisation event [26] and so this is probably a reflection of duplication at the assembly stage (i.e. retention of allelic copies of loci because of the high level of heterozygosity in UK populations). (PDF 1365 kb) [file 13059_2016_985_MOESM1_ESM.pdf]

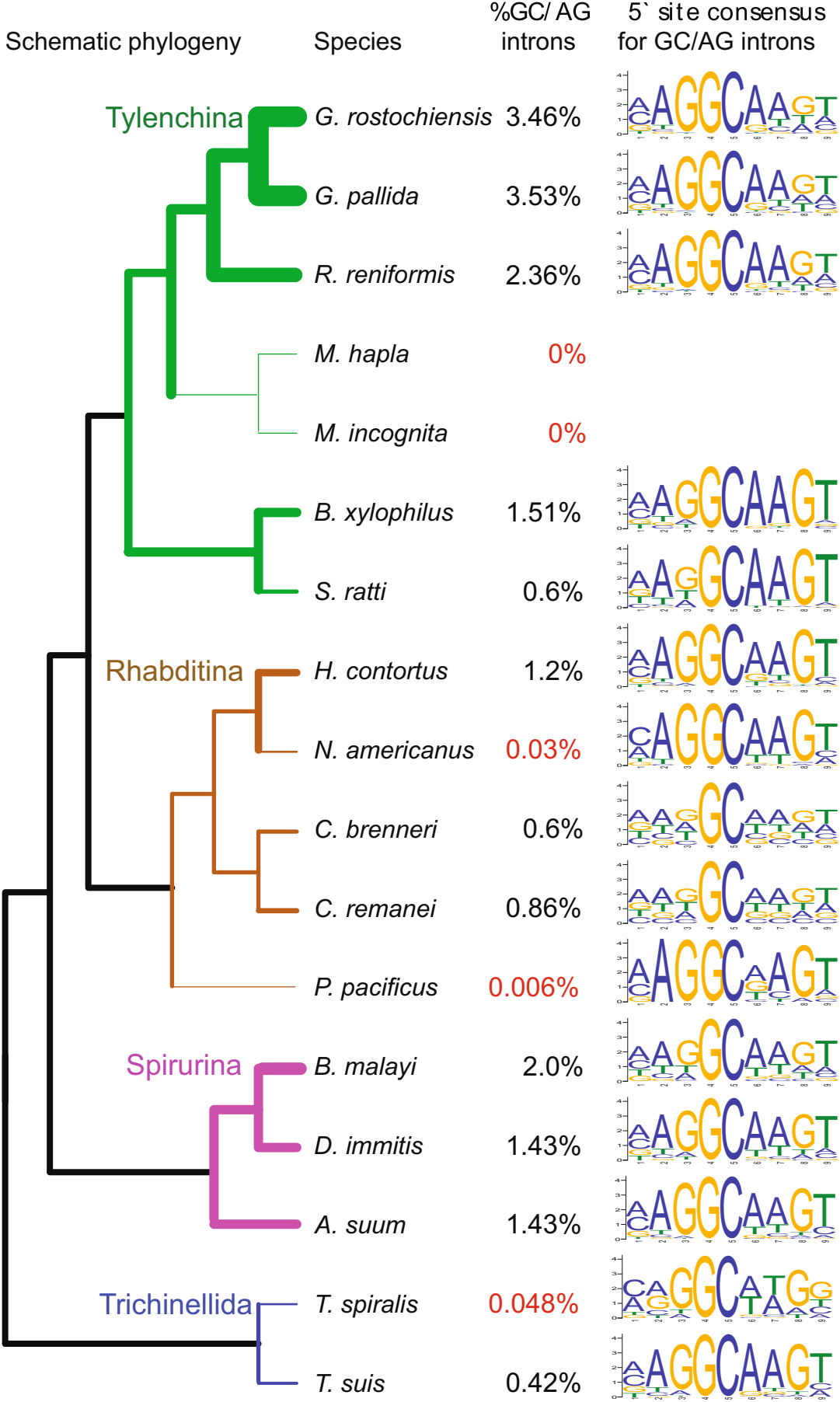

Supplement: Additional file 4: Figure S2. — Phylum wide analysis of GC/AG splice sites in nematodes. The percentage of GC/AG splices sites with associated consensus sequences are shown for 17 species against a schematic phylogeny of the phylum Nematoda (adapted from [89]). Red numbers indicate those which likely represent under reporting due to over-strict parameter settings during gene prediction. (PDF 1371 kb) [file 13059_2016_985_MOESM4_ESM.pdf]

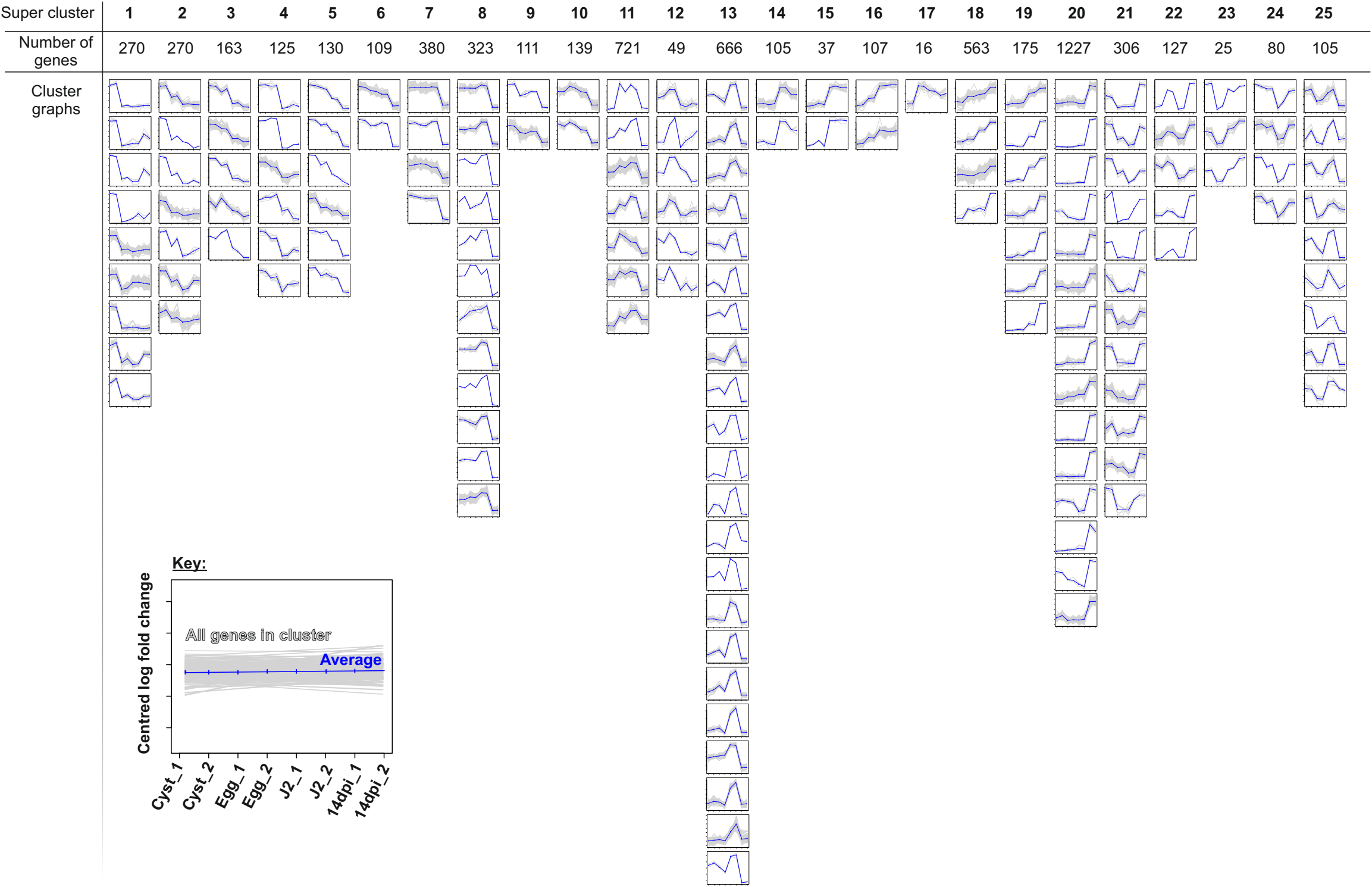

Supplement: Additional file 6: Figure S4. — Differential expression super-clusters. Ninety-four percent of all differentially expressed genes are manually grouped into 25 biologically relevant expression super-clusters. For each super-cluster, individual cluster graphs are shown where for all expression displayed as centred log fold-change is in the order, Cyst, Cyst, Egg, Egg, J2, J2, 14 dpi, 14 dpi. (PDF 1669 kb) [file 13059_2016_985_MOESM6_ESM.pdf]

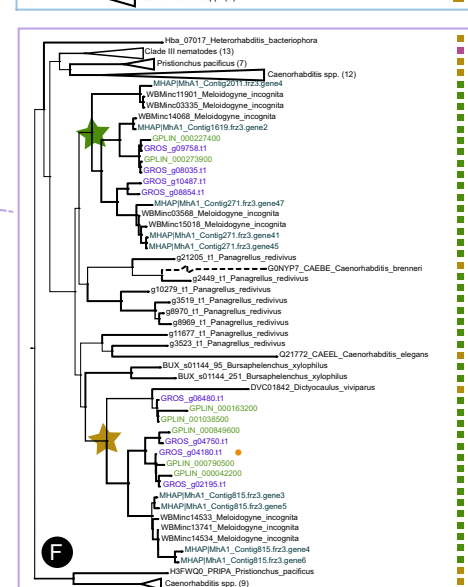

Supplement: Additional file 8: Figure S5. — Phylogenetic analysis of Worm-specific Argonauts (WAGOs). A. A total of 604 nematode and non-nematode argonaute proteins drawn as an unrooted phylogram. It is assumed that each subtree (B–F) is effectively rooted by the other subtrees; however, the extreme divergence between these proteins yields low support for some subtrees. B–F Subtrees containing G. rostochiensis WAGOs (GrWAGOs). Branch widths of subtrees are drawn proportional to branch support. Coloured boxes indicate membership of taxa to phylogenetic groups (nematode Clade I, Clade III, Clade IV and Clade V, non-nematode taxa) and coloured stars indicate clades composed entirely of Heteroderidae-WAGOs (Globodera spp., Meloidogyne spp., Heterodera glycines). Orange dots indicate GrWAGOs in differential expression super-clusters 19, 20 or 21. GrWAGOs are placed within Nematode WAGO-subclades ALG1/ALG2 (1), RDE1/ERGO1/PRG1/2 (2), WAGO1/2/4/5 (7), CSR/WAGO-III/WAGO-IV (8) and NRDE/WAGO-10/11 (5), sensu Buck and Blaxter, 2013 [30]. As expected, no GrWAGOs were observed in WAGO-subclades SAGO2/PPW, WAGO-III/-V and ALG3/ALG4. Subclades NRDE/WAGO-10/11, WAGO1/2/4/5 and CSR/WAGO-III/WAGO-IV show increased numbers of paralogous expansion of these gene families within Clade IV in general and Heteroderidae in particular. B All Globodera spp. and Meloidogyne spp. exhibit one ALG1/ALG2 orthologue each, which form a clade. C The RDE1/ERGO1/PRG1/2 subtree contains another Heteroderidae-specific clade; however, one M. incognita sequence is sister to a subclade of Clade III taxa. This is surprising since the Clade III parasites A. suum and B. pahangi are thought to have lost the piRNA pathway [30] and may be an artefact. D The NRDE/WAGO-10/WAGO-11 subtree shows an expansion of paralogous Heteroderidae-WAGOs, with three GrWAGOs expressed at 14 dpi. E The WAGO1/2/4/5 subtree depicts two Heterodera-specific expansions, of which the larger subclade contains four GrWAGOs expressed at 14 dpi. F The CSR/WAGO-III/WAGO-IV subtree cont [file 13059_2016_985_MOESM8_ESM.pdf]

A. DNA

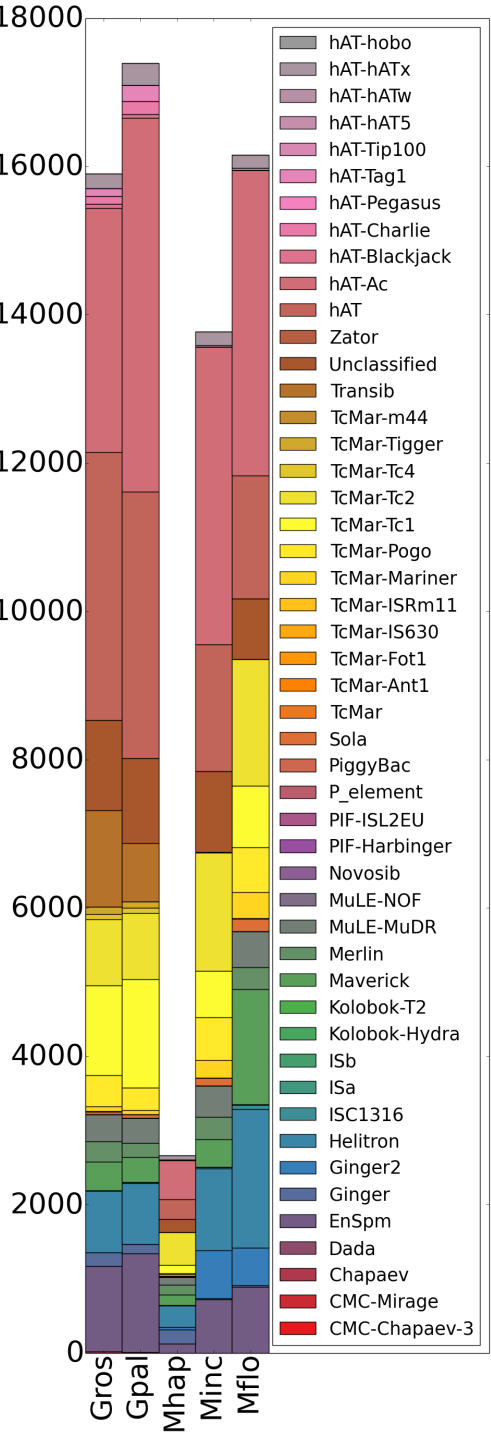

B. LTR

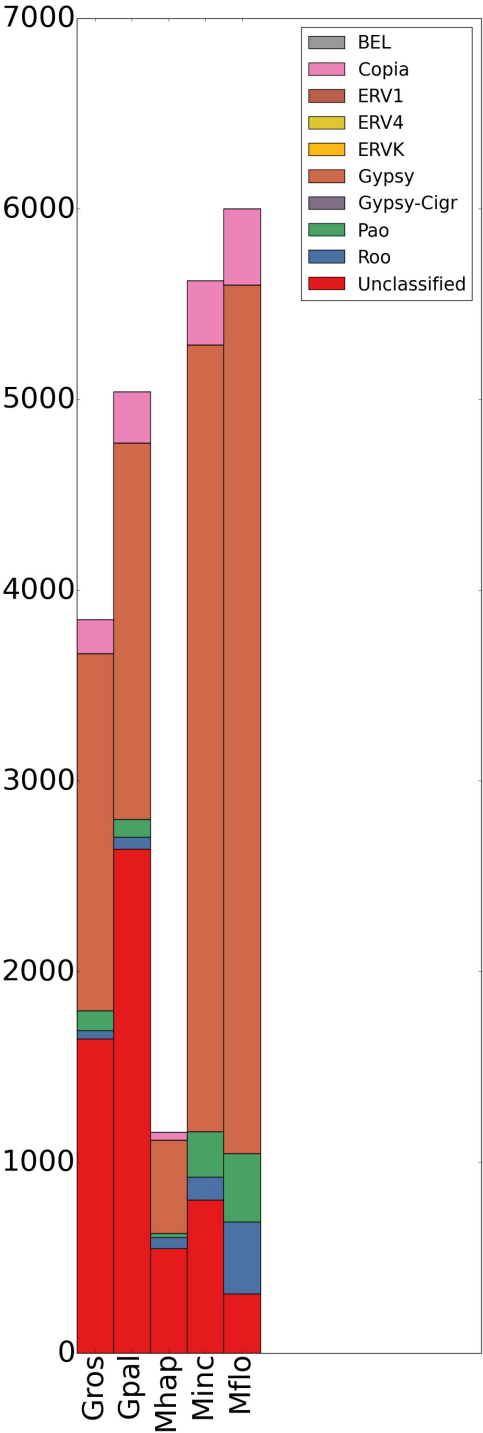

C. LINE

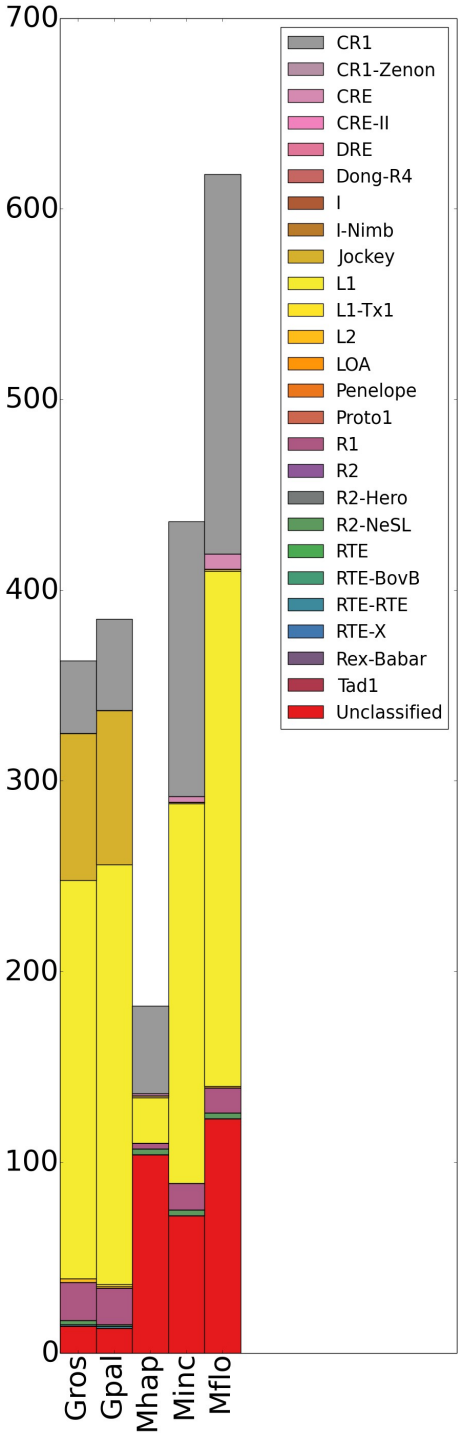

D. SINE

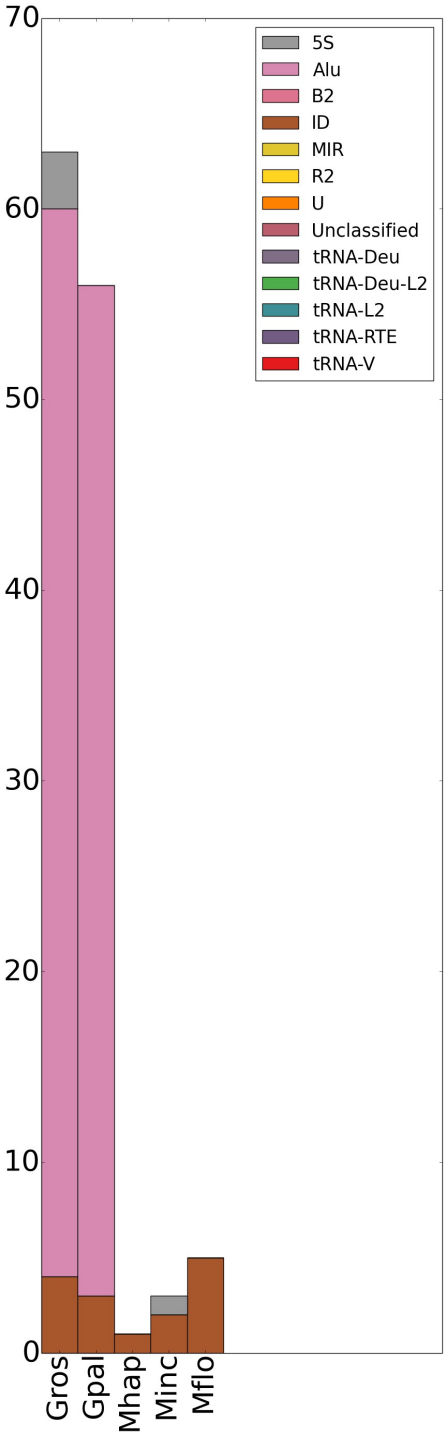

Supplement: Additional file 11: Figure S6. — Transposable elements in G. rostochiensis, G. pallida and Meloidogyne spp. Quantities of transposable elements from (A) DNA, (B) LTR, (C) LINE and (D) SINE super-familes. Globodera spp. contain notably more Jockey (LINE) and Alu (SINE) than the Meloidogyne spp. (PDF 623 kb) [file 13059_2016_985_MOESM11_ESM.pdf]

**A**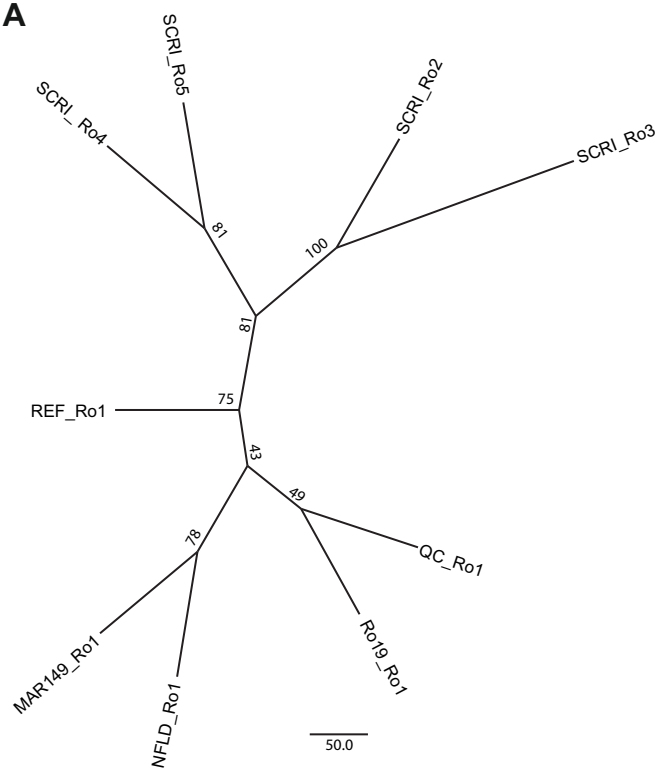**B**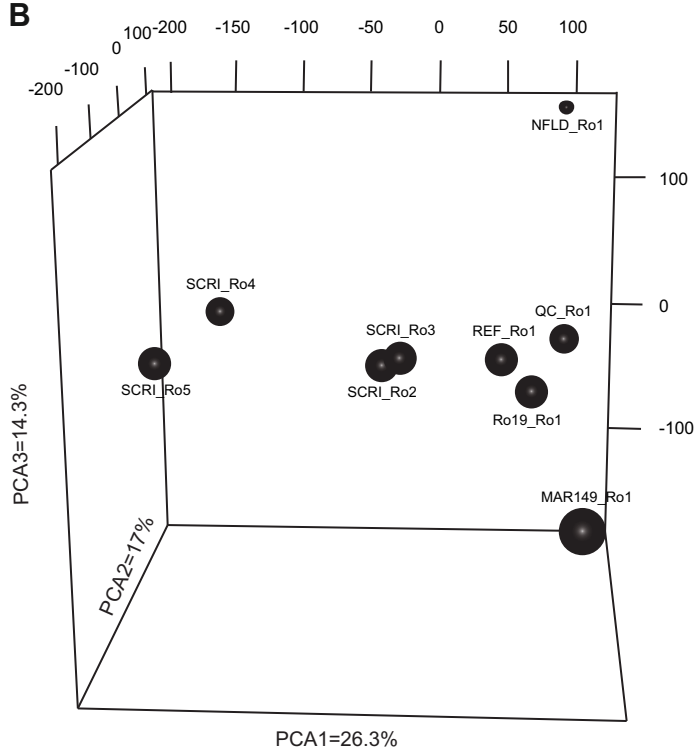

Supplement: Additional file 15: Figure S7. — Phylogenetic analysis of G. rostochiensis pathotypes. A. A maximum likelihood phylogeny based on 730,705 genome wide SNPs. Two distinct groups of Ro1 are together separated from Ro2, Ro3, Ro4 and Ro5. Node labels indicate bootstrap support values for 100 iterations. B. Principle component analysis (PCA) based on the same dataset suggest that for pathotype Ro1, intra-pathotype variation is similar to inter-pathotype variation. REF Ro1 = Reference strain Ro1 assembly, MAR149 Ro1 = British Columbia, QC Ro1 = Quebec, Ro19 Ro1 = European, NFLD Ro1 = Newfoundland, SCRI_Ro2, SCRI_Ro3, SCRI_Ro4, SCRI_Ro5 are pathotype populations from the James Hutton Institute collection. (PDF 1636 kb) [file 13059_2016_985_MOESM15_ESM.pdf]

**A**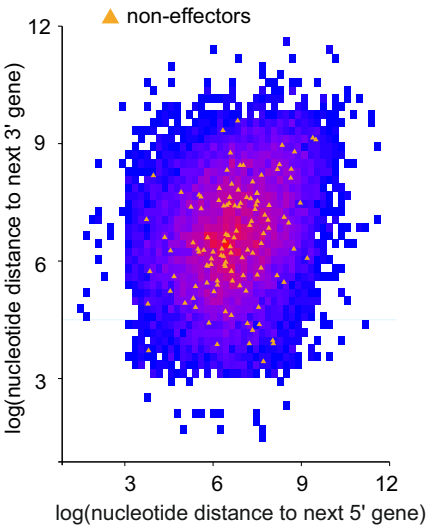**B**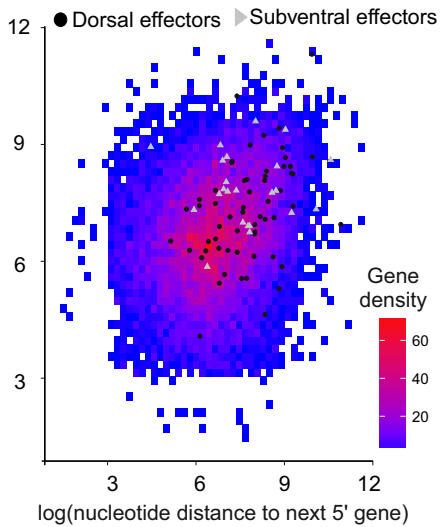**C**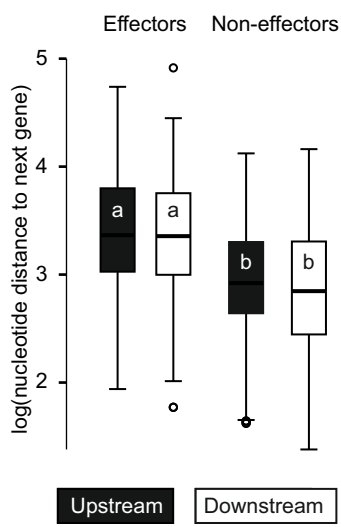

Supplement: Additional file 17: Figure S8. — Comparison of distance to neighbouring gene for effectors and non-effectors. A. The log nucleotide distance to next gene 5′ and 3′ for a random subset of non-effectors (n = 138). B. The log nucleotide distance to next gene 5′ and 3′ for high-confidence effectors (n = 138). C. Comparing the distance of each gene to its neighbour either upstream or downstream suggests that despite being located in gene dense regions of the genome, effectors have a skewed distribution of gene density compared to an identically sized subset of non-effectors (lower case letters indicate homogenous subsets, Student’s t-test correcting for multiple comparisons p <0.001). (PDF 1393 kb) [file 13059_2016_985_MOESM17_ESM.pdf]

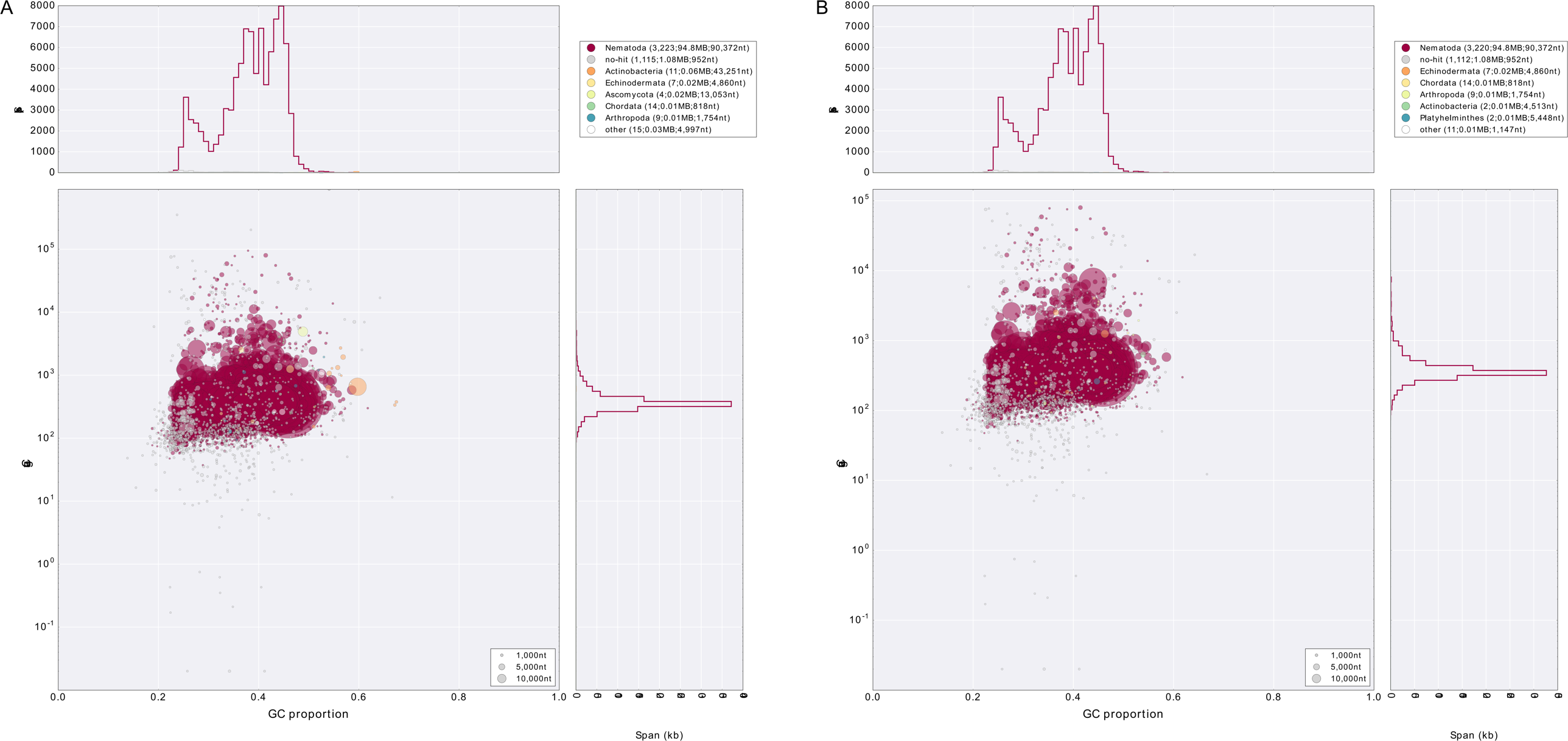

Supplement: Additional file 24: Figure S11. — Blob plots. A. Blob plot of the initial G. rostochiensis assembly, displaying some minor contamination from Actinobacteria and Ascomycota. Each scaffold is drawn as a circle based on its GC content (X-axis) and log-coverage (Y-axis), with a diameter proportional to its length and coloured by its taxonomic annotation at the phylum-level. In the legend, colours of phyla are listed together with scaffold-count, scaffold span and scaffold N50. The histograms above and to the right of the main scatter plot sum contig spans for GC proportion bins and log-coverage bins, respectively. B. Blob plot of the G. rostochiensis assembly after removal of contaminant scaffolds. (PDF 8314 kb) [file 13059_2016_985_MOESM24_ESM.pdf]
