# Supplementary material for: The genome of the yellow potato cyst nematode, Globodera rostochiensis, reveals insights into the basis of parasitism and virulence
Source: Genome Biol. 2016 Jun 10;17:124. doi: 10.1186/s13059-016-0985-1 (PMC4901422; doi:10.1186/s13059-016-0985-1)
Supplement: Additional file 2: — Supplementary methods and results. (DOCX 28 kb) [file 13059_2016_985_MOESM2_ESM.docx]

**SUPPLEMENTARY METHODS AND RESULTS:**

**Library preparation and sequencing**

Two types of library were prepared, a short insert and two mate-pair (Table S11). An amplification-free 400 - 550 bp paired end Illumina library was prepared using a protocol based on a previously described method [1], with the exception of Agencourt AMPure XP beads for sample clean up and size selection. DNA was precipitated onto beads after each enzymatic stage with an equal volume of 20% Polyethylene Glycol 6000 and 2.5 M Sodium Chloride solution. Beads were not separated from the sample throughout the process until after the adapter ligation stage: fresh beads were then used for size selection. 3 kb libraries were constructed following the methods described by Park et al [2].

Libraries were denatured using 0.1 M sodium hydroxide and diluted to 8 pM in hybridisation buffer to allow the template strands to hybridise to adapters attached to the flow cell surface. Cluster amplification was performed on the Illumina cBOT using the V3 cluster generation kit following the manufacturer’s protocol, followed by a SYBRGreen cluster density QC prior to sequencing. DNA on flow cells passing QC were linearized, blocked and hybridized to the R1 sequencing primer. The hybridized flow cells were loaded onto the Illumina HiSeq 2000 for 100 cycles of sequencing-by-synthesis using the V3 SBS sequencing kit then, *in situ*, the linearization, blocking and hybridization step was repeated to regenerate clusters, release the second strand for sequencing and to hybridise the R2 sequencing primer followed by another 100 cycles of sequencing to produce paired-end reads. These steps were performed using proprietary reagents according to manufacturer's recommended protocol (<https://icom.illumina.com/>). Data was analysed from the Illumina HiSeq sequencing machines using the RTA1.8 analysis pipelines.

**Micro satellite prediction**

Genomic scaffolds of *G. rostochiensis* (nGr.v1.0) and *G. pallida* (Gpal.v1.0) were scanned for microsatellite content using the software MSATCOMMANDER [3]. Detection criteria were constrained to perfect repeat motifs of 1-6 bp and a minimum repeat number of 12, 8, 5, 5, 5 and 5, for mono-, di-, tri-, tetra-, penta- and hexa-nucleotide microsatellites, respectively. In order to allow direct comparisons with other nematode species, we used the same criteria than those used by [4] to search for microsatellite array in *M. incognita*, *M. hapla*, *C. elegans*, *P. pacificus* and *B. malayi*. Repeat content in the two genomes was largely similar, but differed from, for example, *Meloidogyne* species in the number and pattern of microsatellite repeats. *G. rostochiensis* had 20,015 microsatellite loci, and *G. pallida* 22,627, but *M. incognita* had only 4880 and *M. hapla* 2842 [4]. While *G. rostochiensis* contained in frequency more mono-nucleotide microsatellites, *G. pallida* had more tetra- and penta-nucleotide microsatellites (Table S12, *χ*^2^ test of independence, p < 0.0001).

**Transposable element prediction**

The results of three transposable element (TE) prediction methods were combined to mitigate biases. One was DNA sequence homology based using reference DNA sequences of TEs in a *de novo* constructed library, representing our genome assembly, as well as that of *G. pallida [5], Meloidogyne hapla* [6], *M. incognita* [7] and *M. floridensis* [8]. RepeatModeler 1.0.4 [9] was used to identify repeat sequences in each genome assembly using RECON [10], RepeatScout [11] and TRF [12]. RepeatModeler uses RepeatMasker [13] to classify the consensus sequences of the recovered repetitive sequence clusters. The identification stage employed RMBlast [14] and the Eukaryota TE library from Repbase Update [15]. The uclust algorithm in USEARCH [16] was used to make a nonredundant library, picking one representative sequence for each 80% identical cluster. Additional classification of the consensus sequences was performed with the online version of Censor [17]. Classifications supported by matches with a score value larger than 300 and 80% identity were retained. Once ready, the library was used in RepeatMasker to search for repeat sequences in the genome assemblies. To eliminate redundancies in RepeatMasker output, we used One Code to Find Them All [18], which assembled overlapping matches with similar classifications, and retained only the highest scoring match of any remaining group of overlapping matches. Alternative approaches to identify TEs were TransposonPSI (http://transposonpsi.sourceforge.net/), which searches for protein sequence matches in a protein database thus allowing accurate identification of shorter fragments, and LTRharvest [19], which identifies secondary structures. For TransposonPSI searches, only chains with a combined score larger than 80 were retained, while we retained only matches that were at least 2000 bp long and 80% similar to the query from LTRharvest searches. Where matches from the three approaches overlapped, we retained only the longest match. Python wrappers to execute this workflow are available at https://github.com/HullUni-bioinformatics/TE-search-tools. TE assemblage in *G. rostochiensis* is dominated by “cut and paste” DNA elements, with hAT and TcMar families most abundant. Globodera TEs appear to diverge from those of other tylenchomorph species, mostly in the presence of Jockey and Alu families (Figure S5).

**Gene prediction**

*De novo* gene prediction on assembly nGr.v0.9 was carried out by constructing Snap (version 2006-07-28) HMMs (based on Cegma output) and Genemark (v2.4) HMMs, which were supplied to MAKER2 (v2.31), together with an RSEM’d Trinity assembly (default parameters [20]) as EST evidence and *G. pallida* proteins [5] as protein homology evidence. The resulting GFF3 file was used to train and run AUGUSTUS (v3.0.3), resulting in annotation nGr.v0.9.auto containing 13,650 gene models. Using the collaborative genome annotation editor WebApollo, approximately 1/8^th^ of the gene models were manually inspected based on homology to known nematode genes, RNAseq evidence and WGS read coverage yielding 1,566 manually curated gene models (nGr.v0.9.manual). In a second round of *de novo* gene prediction, the gap-filled assembly nGr.v1.0 was used for SNAP HMM and Genemark HMM construction and provided to MAKER2 in addition to RSEM’d Trinity assembly as “EST evidence” and the set of all full-length proteins in nGr.v0.9.auto and Gr.v0.9.manual as protein homology evidence. RNAseq reads were mapped to the genome using GSNAP (v2014-12-17) in order to generate an intron-hints file (following the workflow described in http://bioinf.uni-greifswald.de/bioinf/wiki/pmwiki.php?n=IncorporatingRNAseq.GSNAP). The MAKER2 annotation was used, together with the RSEM’d Trinity assembly and the intron-hints file, to retrain and run Augustus, which generated the final gene set nGr.v1.0 containing 14,309 proteins. This final gene prediction set improved upon automated predictions; as evidenced by an increase in the number of sequences containing Pfam domains, the number of sequences which have Nematode sequences as their best blast hit against nr (BLASTp , Evalue 1e-10), and the number of well supported non-canonical splice sites (Table S1). In the final set of 14,378 genes, ~95% had evidence of transcription in the life stages sampled (cumulative FPKM>1; see below).

**REFERENCES:**

1. Kozarewa I, Ning Z, Quail MA, Sanders MJ, Berriman M, Turner DJ: **Amplification-free Illumina sequencing-library preparation facilitates improved mapping and assembly of (G+ C)-biased genomes.** *Nature methods* 2009, **6:**291-295.

2. Park N, Shirley L, Gu Y, Keane TM, Swerdlow H, Quail MA: **An improved approach to mate-paired library preparation for Illumina sequencing.** *Methods in Next Generation Sequencing* 2013, **1:**10-20.

3. Faircloth BC: **msatcommander: detection of microsatellite repeat arrays and automated, locus‐specific primer design.** *Molecular Ecology Resources* 2008, **8:**92-94.

4. Castagnone-Sereno P, Danchin EG, Deleury E, Guillemaud T, Malausa T, Abad P: **Genome-wide survey and analysis of microsatellites in nematodes, with a focus on the plant-parasitic species Meloidogyne incognita.** *BMC genomics* 2010, **11:**598.

5. Cotton JA, Lilley CJ, Jones LM, Kikuchi T, Reid AJ, Thorpe P, Tsai IJ, Beasley H, Blok V, Cock PJ: **The genome and life-stage specific transcriptomes of *Globodera pallida* elucidate key aspects of plant parasitism by a cyst nematode.** *Genome Biology* 2014, **15:**R43.

6. Opperman CH, Bird DM, Williamson VM, Rokhsar DS, Burke M, Cohn J, Cromer J, Diener S, Gajan J, Graham S: **Sequence and genetic map of *Meloidogyne hapla*: A compact nematode genome for plant parasitism.** *Proceedings of the National Academy of Sciences* 2008, **105:**14802-14807.

7. Abad P, Gouzy J, Aury J-M, Castagnone-Sereno P, Danchin EGJ, Deleury E, Perfus-Barbeoch L, Anthouard V, Artiguenave F, Blok VC, et al: **Genome sequence of the metazoan plant-parasitic nematode *Meloidogyne incognita*.** *Nature Biotechnology* 2008, **26:**909-915.

8. Lunt DH, Kumar S, Koutsovoulos G, Blaxter ML: **The complex hybrid origins of the root knot nematodes revealed through comparative genomics.** *PeerJ* 2014, **2:**e356.

9. Smit A, Hubley R: **RepeatModeler Open-1.0.** *Repeat Masker Website* 2010.

10. Bao Z, Eddy SR: **Automated de novo identification of repeat sequence families in sequenced genomes.** *Genome Research* 2002, **12:**1269-1276.

11. Price AL, Jones NC, Pevzner PA: **De novo identification of repeat families in large genomes.** *Bioinformatics* 2005, **21:**i351-i358.

12. Benson G: **Tandem repeats finder: a program to analyze DNA sequences.** *Nucleic acids research* 1999, **27:**573.

13. Smit AF, Hubley R, Green P: **RepeatMasker Open-3.0.** 1996.

14. Camacho C, Coulouris G, Avagyan V, Ma N, Papadopoulos J, Bealer K, Madden TL: **BLAST+: architecture and applications.** *BMC bioinformatics* 2009, **10:**421.

15. Jurka J, Kapitonov VV, Pavlicek A, Klonowski P, Kohany O, Walichiewicz J: **Repbase Update, a database of eukaryotic repetitive elements.** *Cytogenetic and genome research* 2005, **110:**462-467.

16. Edgar RC: **Search and clustering orders of magnitude faster than BLAST.** *Bioinformatics* 2010, **26:**2460-2461.

17. Jurka J, Klonowski P, Dagman V, Pelton P: **CENSOR—a program for identification and elimination of repetitive elements from DNA sequences.** *Comput Chem* 1996, **20:**119-121.

18. Bailly-Bechet M, Haudry A, Lerat E: **“One code to find them all”: a perl tool to conveniently parse RepeatMasker output files.** *Mob DNA* 2014, **5:**13.

19. Ellinghaus D, Kurtz S, Willhoeft U: **LTRharvest, an efficient and flexible software for de novo detection of LTR retrotransposons.** *BMC bioinformatics* 2008, **9:**18.

20. Grabherr MG, Haas BJ, Yassour M, Levin JZ, Thompson DA, Amit I, Adiconis X, Fan L, Raychowdhury R, Zeng Q, et al: **Full-length transcriptome assembly from RNA-Seq data without a reference genome.** *Nature Biotechnology* 2011, **29:**644-U130.
