# Supplementary material for: The genome of the yellow potato cyst nematode, Globodera rostochiensis, reveals insights into the basis of parasitism and virulence
Source: Genome Biol. 2016 Jun 10;17:124. doi: 10.1186/s13059-016-0985-1 (PMC4901422; doi:10.1186/s13059-016-0985-1)

A Correlate expression of **all** genes between stages

stage	J2	7	14	21
J2	1	0.44	0.55	0.13
7		1	**0.84**	0.42
14			1	**0.60**
21				1

J2 = 2nd stage juvenile
7,14 or 21 = days post infection

Key

1
0.9
0.8
0.7
0.6
0.5
0.4
0.3
0.2
0.1
0

B Correlate expression of **effector** genes between stages

stage	J2	7	14	21
J2	1	-0.07	-0.07	-0.04
7		1	**0.89**	0.46
14			1	**0.65**
21				1

J2 = 2nd stage juvenile
7,14 or 21 = days post infection

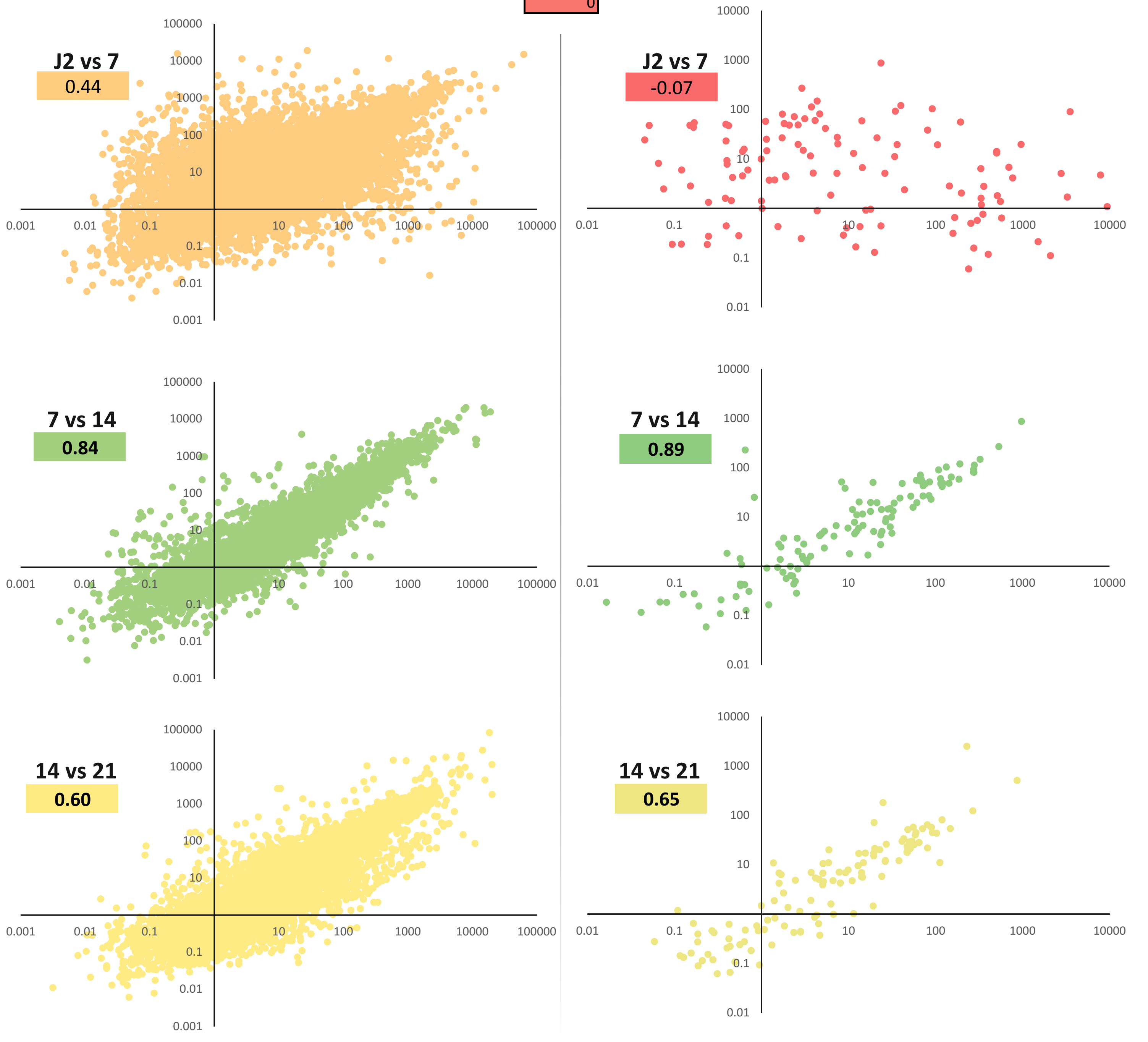

Supplement: Additional file 5: Figure S3. — Comparison of gene expression between parasitic G. pallida life stages. The key transitions to be captured in terms of gene expression of all genes, and in particular for effectors, is from outside the plant (J2) compared to inside the plant (sedentary female). There is almost no difference in global gene expression between the early sedentary time points [22]. A. 84 % of the variation in expression at 7 days post infection (dpi) is explained by variation in expression at 14 dpi. B. This correlation is even more profound if the analysis is restricted to only the effectors (89 %). Similar correlations are possible between 14 and 21 dpi, albeit of lesser magnitude but an identical trend (60 % correlation for all genes, and 64 % correlations for specifically effectors). This is not the case, however, when comparing J2 and 7 dpi (44 % for all genes, and zero correlation for all effectors). Fourteen dpi provides an ideal intermediate for the sedentary stages. (PDF 4379 kb) [file 13059_2016_985_MOESM5_ESM.pdf]
