# Supplementary material for: The genome of the yellow potato cyst nematode, Globodera rostochiensis, reveals insights into the basis of parasitism and virulence
Source: Genome Biol. 2016 Jun 10;17:124. doi: 10.1186/s13059-016-0985-1 (PMC4901422; doi:10.1186/s13059-016-0985-1)
Supplement: Additional file 20: Figure S10. — The DOG box is not a quantitative predictor of temporal expression. A. In G. pallida, genes with four or more motifs and a signal peptide are primarily expressed during the infective stage. Each line represents the expression pattern of a different gene. B. The number of motifs does not correlate with gene expression at J2 (black, all numbers of motifs; red, four or more motifs). (PDF 1707 kb) [file 13059_2016_985_MOESM20_ESM.pdf]

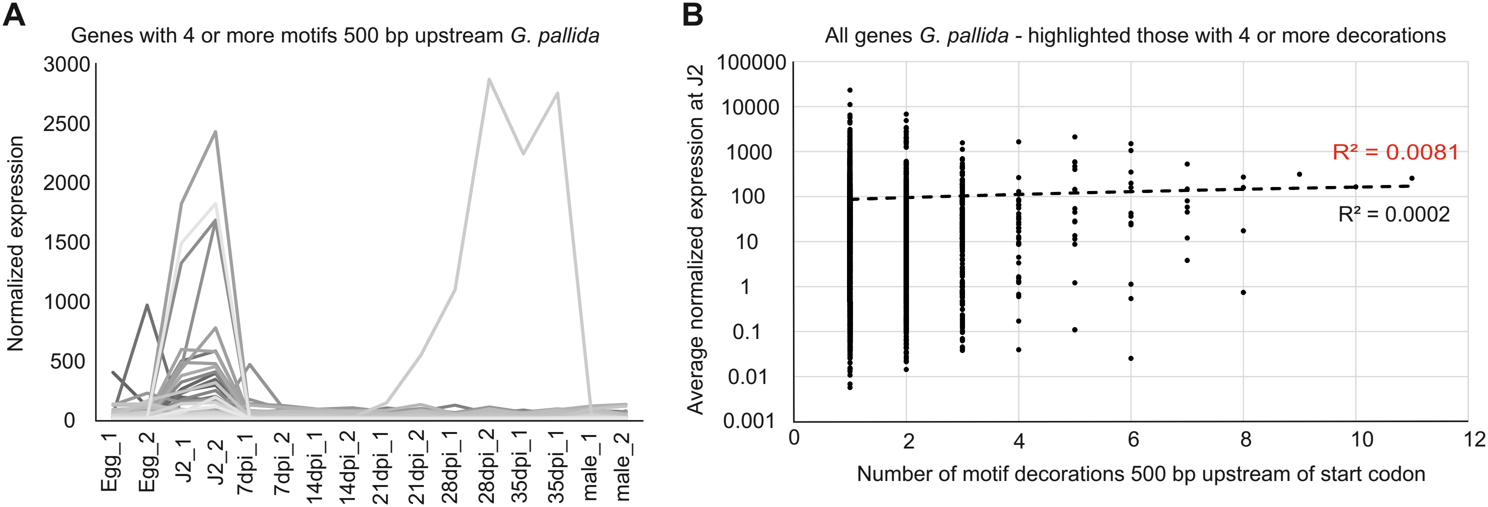
A
Genes with 4 or more motifs 500 bp upstream G. pallida
Normalized expression
3000
2500
2000
1500
1000
500
0
Egg_1
Egg_2
J2_1
J2_2
7dpi_1
7dpi_2
14dpi_1
14dpi_2
21dpi_1
21dpi_2
28dpi_1
28dpi_2
35dpi_1
35dpi_1
male_1
male_2
B
All genes G. pallida - highlighted those with 4 or more decorations
Average normalized expression at J2
100000
10000
1000
100
10
1
0.1
0.01
0.001
R² = 0.0081
R² = 0.0002
0
2
4
6
8
10
12
Number of motif decorations 500 bp upstream of start codon
