# Supplementary material for: The genome of the yellow potato cyst nematode, Globodera rostochiensis, reveals insights into the basis of parasitism and virulence
Source: Genome Biol. 2016 Jun 10;17:124. doi: 10.1186/s13059-016-0985-1 (PMC4901422; doi:10.1186/s13059-016-0985-1)

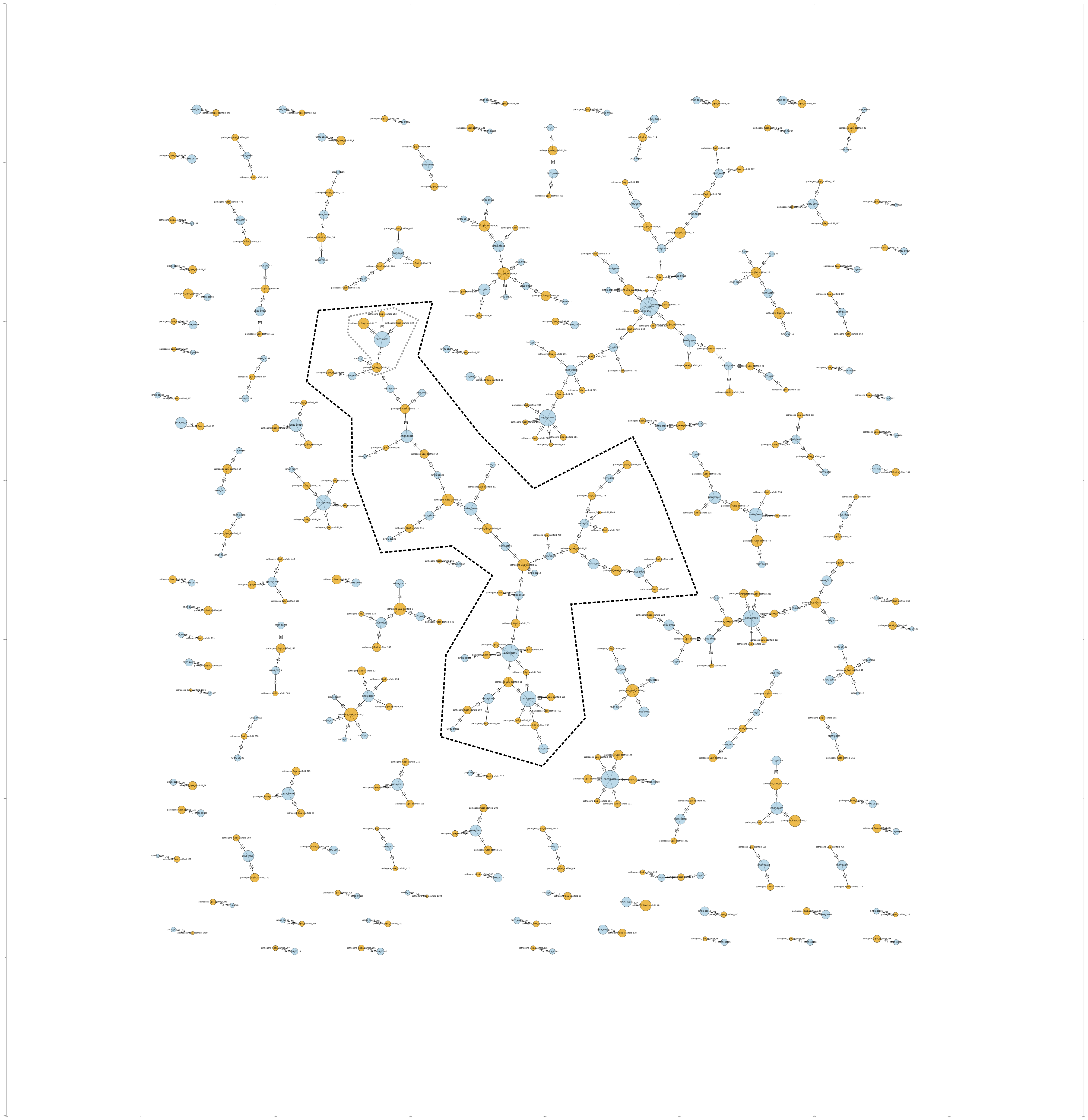

Supplement: Additional file 19: Figure S9. — Synteny clusters. Network representation of i-ADHoRe results. Scaffolds are represented as nodes (blue = G. rostochiensis, ochre = G. pallida) whose diameter is scaled by scaffold length and vertices are drawn between them if five or more anchorpoints (i.e. syntenic proteins, numbers on vertices indicate count of anchorpoints) have been found. The biggest cluster (dashed black line) is composed of 26 G. rostochiensis scaffolds (4.99 Mb) and 35 G. pallida scaffolds (5.54 Mb). Part of this cluster (grey dotted line) is shown in Fig. 6. (PDF 570 kb) [file 13059_2016_985_MOESM19_ESM.pdf]
